# Composition tunability of semiconductor radiosensitizers for low-dose X-ray induced photodynamic therapy

**DOI:** 10.1186/s12951-022-01494-7

**Published:** 2022-06-21

**Authors:** Lei Chen, Jinghui Zhang, Lihua Xu, Luchao Zhu, Jinpeng Jing, Yushuo Feng, Zongzhang Wang, Peifei Liu, Wenjing Sun, Xiangmei Liu, Yimin Li, Hongmin Chen

**Affiliations:** 1 State Key Laboratory of Molecular Vaccinology and Molecular, Diagnostics & Center for Molecular Imaging and Translational Medicine, School of Public Health, Xiamen University, Xiamen, 361102 People’s Republic of China; 2 State Key Laboratory of Organic Electronics and Information Displays & Institute of Advanced Materials (IAM), Jiangsu Key Laboratory for Biosensors, Nanjing University of Posts & Telecommunications, Nanjing, 210023 People’s Republic of China; 3 ZJU-Hangzhou Global Scientific and Technological Innovation Center, Hangzhou, 311200 People’s Republic of China; 4 Department of Radiation Oncology, Cancer Center, The First Affiliated Hospital of Xiamen University, Xiamen, 361003 People’s Republic of China; 5 The Third Clinical Medical College, Fujian Medical University, Fuzhou, People’s Republic of China

**Keywords:** Radiosensitizers, Semiconductors, Reactive oxygen species, Composition tunability, X-ray induced photodynamic therapy

## Abstract

Radiation therapy is one of the most commonly used methods in clinical cancer treatment, and radiosensitizers could achieve enhanced therapeutic efficacy by incorporating heavy elements into structures. However, the secondary excitation of these high-Z elements-doped nanosensitizers still imply intrinsic defects of low efficiency. Herein, we designed Bi-doped titanium dioxide nanosensitizers in which high-Z Bi ions with adjustable valence state (Bi^3+^ or Bi^4+^) replaced some positions of Ti^4+^ of anatase TiO_2_, increasing both X-rays absorption and oxygen vacancies. The as-prepared TiO_2_:Bi nanosensitizers indicated high ionizing radiation energy-transfer efficiency and photocatalytic activity, resulting in efficient electron–hole pair separation and reactive oxygen species production. After further modification with cancer cell targeting peptide, the obtained nanoplatform demonstrated good performance in U87MG cell uptakes and intracellular radicals-generation, severely damaging the vital subcellular organs of U87MG cells, such as mitochondrion, membrane lipid, and nuclei etc*.* These combined therapeutic actions mediated by the composition-tunable nanosensitizers significantly inhibited the U87MG tumor growth, providing a refreshing strategy for X-ray induced dynamic therapy of malignant tumors.

**Supplementary Information:**

The online version contains supplementary material available at 10.1186/s12951-022-01494-7.

## Introduction

Radiation therapy (RT) is a regular strategy to treat cancer in clinic [1, 2]. Due to high irradiation, normal tissues might be inevitably damaged [3, 4]. To lower the side effect and enrich the therapeutic efficacy, recent researches focused on the construction of nanoscintilators to achieve X-ray excited photodynamic therapy (X-PDT), which overcomes the limitation of penetration depth in tissues of PDT and enhances the efficacy of RT under low-dose irradiation [5, 6]. Generally, nanoscintillators are firstly excited by the X-rays to emit X-ray excited luminescence (XEOL), and then the XEOL energies activate the dynamic reaction of photosensitizer through a fluorescence resonance energy transfer (FRET) [7, 8]. However, the indirect energy transfer process causes energies losses [9, 10]. X-PDT is a therapy methodology combining photodynamic therapy (PDT) and radiation therapy (RT). There are plenty of problems with X-PDT such as weak therapeutic efficacy and radioresistance of X-ray-activated therapies, which is induced by hypoxia in tumor [11, 12]. So, it is urgent to develop nanosensitizers that could achieve efficient inhibition of the tumor growth under hypoxia [13, 14].

Previous publications have demonstrated that semiconductors (TiO_2_, ZnO, etc*.*) could generate great photoelectrochemical effect under X-ray irradiations, which promotes highly efficient electron–hole pair separation and subsequent reactive oxygen species (ROS) production [15, 16]. The Shi group employed ZnO as the photosensitizer to enhance the type-I PDT induced by X-ray [17]. The emission from nanoscintillators matched well with the absorption of ZnO layers to ensure the energy transfer and generate ROS from the interaction between excitons (i.e., the electron–hole pairs) and water. This unique strategy minimized the dependency of oxygen species, enhancing antitumor efficacy. TiO_2_, as semiconductor with wide band gap, has been applied in radiation oncology. The Sasaki group prepared polyacrylic acid-modified titanium peroxide nanoparticles (PAA-TiOxNPs) from anatase TiO_2_, which revealed favorable radiation-enhancement effect on pancreatic cancer [18]. However, due to the low X-ray energy deposition, high dosage still need to be employed to kill cancer cells [19, 20].

High-Z elements, such as Hf, have been proved radio-enhancement in clinical trials (NCT04505267, NCT04484909) [21]. The Dai group reported a Hf-polyphenolic chelate-based nanosensitizer with positive modulation capability of radiosensitization and H_2_S-based oxygenation [22]. By integrating H_2_S-reprogrammed oxygen metabolism with Hf-sensitized radiotherapy, this design achieved both primary tumor eradication and immune activation against distal tumors. Bismuth (Bi) with K edge energy at 90 keV, that is over that of Hf at 65 keV, is the ideal metal element for constructing radiosensitizers [23, 24]. The Zhao group synthesized BSA coated BiOI@Bi_2_S_3_ heterojunction nanoparticles using anion exchange method. BiOI@Bi_2_S_3_ NPs can be excited by X-ray to eject photoelectrons which transfer from the conduction band of Bi_2_S_3_ to that of BiOI, while the holes move in the opposite direction [25]. The efficient separation of electrons and holes improved the generation of ROS via photocatalytic process, leading to highly efficient radiosensitization and photosensitization. We hypothesized that composition tunability of high-Z elements in semiconductors could achieve more efficient nano-radiosensitizers.

Herein, we prepared Bi-doped semiconductors TiO_2_ (TiO_2_:Bi, TB) nanoparticles for tumor ablation under low-dose irradiation. Due to high-Z elements doping, the TB possesses a high radiation attenuation ability and increases the deposition of X-ray, which can offer more favorable depth of penetration under X-ray irradiation than that of other TiO_2_-based materials under UV irradiation [26]. More intriguingly, Bi (Z = 83) has higher absorption coefficient than that of Au and Pt. Compared with other high-Z elements (Au and Pt) doped TiO_2_-based materials, TB can improve X-ray absorption efficiency, and enhance the production capacity of reactive oxygen species with lower X-ray dose [18, 27]. Our results showed that Bi ions (Bi^3+^ or Bi^4+^) are easy to replace the position of Ti^4+^ in anatase structures, as the size of Bi ions (Bi^3+^ or Bi^4+^) is closed to Ti^4+^[28]. The incorporation of Bi (i.e., Bi^3+^ and Bi^4+^) enhanced the generation of hydroxyl radical (∙OH). Investigations in vitro and in vivo demonstrated that this composition-tunable TB nanoparticle improved the ROS-generation capability of semiconductors TiO_2_ under low-dose X-ray irradiation and achieved more efficiencies of tumor therapy. Overall, by combining with the great potential of Bi ions in separating the photogenerated electron–hole pairs, the as-designed nanoplatform realized low-dose X-ray excited combination therapies for malignant tumors.

## Materials and methods

### Synthesis of Bi-doped anatase-TiO_2_ nanoparticles (TB)

14.5 mg of Bi(NO_3_)_3_·5H_2_O was dissolved in ethylene glycol (25 mL) at room temperature. Tetrabutyl titanate (1 mL) was added under vigorous stirring. Next, the system was bubbled with nitrogen for about 10 min to remove the oxygen and water. After that, the system was sealed with parafilm and was kept stirring for 24 h. The solution sample was then poured into a mixture of acetone (100 mL) and ultrapure water (1 mL), and reacted for 1 h under vigorous stirring. After being left for 48 h, the white precipitation was harvested by centrifugation (10,000 rpm, 10 min) after another standing reaction for 48 h, followed by washing with ethanol and acetone for four times to remove residual ethylene glycol. The precursor was dried under vacuum for overnight and calcined at 450 °C for 2 h (1 °C/min), getting the Bi-doped anatase-TiO_2_ nanoparticles (TB).

### Surface modification of TB

To obtain the carboxyl-functioned TiO_2_:Bi, the as-prepared TB was dispersed in NaOH solution (pH = 10) and stirred vigorously for overnight. Then, the activated TB was dispersed in 20 mL of anhydrous ethanol under sonication. 45 mg of silane-PEG_3400_-COOH was added and reacted for 12 h at 70 °C under vigorous stirring. The resulting TB-PEG-COOH was collected and washed with ultrapure water and ethanol sequentially for three times.

To further obtain the TiO_2_:Bi modified with RGD (c(RGD)fk), the as-prepared TB-PEG-COOH (100 mg) was re-dispersed in 14 mL of ultrapure water, and then mixed with 2 mmol of EDC [1-(3-Dimethylaminopropyl)-3-ethylcarbodiimide hydro] and NHS (N-Hydroxysuccinimide) The mixture was stirred in the dark for 1 h, followed by addition of c(RGD)fk (5 mg). Subsequently, the mixture was stirred at room temperature in the dark for 12 h, and then washed centrifugally (10,000 rpm, 10 min) for three times with ultrapure water and dried at 60 °C for 2 h, getting the TB-PEG-RGD nanoplatform (TBR). The synthesis method of TR is the same as that of TBR except that Bi(NO_3_)_3_·5H_2_O is not added in the first step.

### Hydroxyl radicals (OH) generation in solution under X-ray irradiation

To detect OH in solution, methylene blue (MB) was used as detection probe. TB was suspended in water (100 µg/mL) in the presence of 20 µM of MB, and then were irradiated by X-ray irradiation at a dose of 4 Gy (50 kV, 70 μA). Free MB or PBS were employed as controls. The characteristic UV–Vis absorption spectra (peak at 662 nm) of MB were measured to indicate the OH-generation.

### The cytotoxicity evaluation

U87MG cells were cultured in Dulbecco’s modified Eagle’s medium (DMEM) medium that contained 10% Fetal bovine serum (FBS) in a humidified atmosphere with 5% CO_2_ at 37 °C. The cells were seeded on 96-well plates (10^4^ cells per well) and incubated for 24 h prior to the experiments. The nanoplatform with different concentrations (0–200 μM) was added into the medium and incubated with U87MG cells for 24 h in dark. The cytotoxicity was evaluated using the standard MTT assay.

### In vitro therapeutic efficacy evaluation

The U87MG cells were incubated with TBR (0–200 µg/mL) for 24 h, and then were irradiated by X-ray (4 Gy). After incubation for another 24 h, the cell viability was determined using the MTT assay.

### Intracellular hydroxyl radical generation

To monitor intracellular OH generation, 2 × 10^5^ U87MG cells were cultured in glass bottom cell culture dish (Ø = 10 mm) for 24 h. Then, TBR (200 μg/mL) was incubated with cells for additional 24 h. Before X-ray irradiation (4 Gy), DCFH-DA (1 μM) were firstly incubated with cells for 30 min. Fluorescence images were acquired on an Olympus FV1200 laser scanning confocal microscope using a FITC filter (Ex/Em: 488/525 nm).

### Flow cytometric analysis

U87MG cells were incubated with TBR (200 μg/mL) for 24 h, and then treated with X-ray irradiation. After another incubation for 24 h, the cells were treated by trypsinization, harvesting, rinsing, and redispersing, and labeled with annexin V-FITC/PI. The apoptosis of U87MG cells was recorded using flow cytometer (Beckman, Cyan ADP).

### Mitochondrial membrane potential measurement

U87MG cells (2 × 10^5^) were seeded into glass bottom cell culture dishes (Ø = 10 mm). TBR (200 μg/mL) was added and incubated with cancer cells for 24 h. After X-ray irradiation (4 Gy), the cells were washed with PBS for three times and then stained with Hoechst 33,342 (5 μg/mL) (Ex/Em: 346/460 nm) and JC-1 dye (5 μM) for 20 min. Fluorescence images were acquired on an Olympus FV1200 laser scanning confocal microscope (JC-1 Aggregates, Ex/Em: 585/590 nm; JC-1 Monomer, Ex/Em: 510/527 nm).

### Intracellular lipid peroxide measurement

U87MG cells (2 × 10^5^) were seeded into glass bottom cell culture dishes (Ø = 10 mm). TBR (200 μg/mL) was added and incubated with cancer cells for 24 h. After X-ray irradiation (4 Gy), the cells were washed with PBS for three times and then stained with Hoechst 33342 (5 μg/mL) and BODIPY C11 (5 μM) for 20 min. The intracellular lipid peroxide was monitored using Olympus FV1200 laser confocal scanning microscope (using a FITC filter. Ex/Em: 488/525 nm) after washing by PBS.

### DNA damage measurement (comet assay)

After different treatments, 10^5^/mL of the treated U87MG cells were mixed with molten LMAgarose (at 37 °C) at a ratio of 1:10 (v/v). 50 μL of the solution was pipetted onto a CometSlide™. The slide was then immersed in lysis solution (4 °C) for overnight. Neutral electrophoresis buffer (1× , 4 °C) was added to the electrophoresis gel box and the slides were placed in a slide tray. The power supply was set at 21 V. After 45 min, the slides were gently removed and immersed in DNA precipitation solution for 30 min, and then in 70% ethanol for 30 min at room temperature. The slides were dried and stained in SYBR^®^ safe for 30 min in the dark. The single cell nucleus images were acquired on an inverted fluorescence microscope.

### Radiosensitization measurement (clonogenic assay)

U87MG cells (2 × 10^5^) were seeded in 6-well plates and cultured for 12 h. After co-incubation with TBR (200 μg/mL) for 24 h, cells were irradiated with X-ray at dosage of 0, 2, 4, 6, 8 and 10 Gy, separately. After another incubation for 24 h, cancer cells were trypsinized and counted immediately. 1000 cells were re-seeded on 6-well plates and cultured in 2 mL of medium for 14 days. When cell populations (> 50 cells) were observed, the culture medium was discarded and the plates washed with PBS for two times. 500 μL of 0.5% crystal violet (dispersed in 50% methanol) was added in each well for staining. The wells were then washed with water and the cell populations were counted. The cell population numbers are converted to the survival fractions by normalizing to the control groups (0 Gy) for each treatment.

According to the survival curves in clonogenic assay, dose required for 10% survival (D10) can be obtained as follow. The radiosensitization is confirmed by fitting the dose–response curves with the function F(D) = exp(− αD − βD^2^), where D is the radiation dose and F(D) is the survival fraction. A greater α/β value indicates a strong early radiation response. Dose enhancement factor (DEF) was calculated as the ratio between RT and X-PDT radiation doses at 10% survival fractions.

### Statistical analysis

All data were presented as mean ± standard deviation. Comparison of the data were conducted with Student’s t-test (*P < 0.05, **P < 0.01, and ***P < 0.001).

## Results and discussion

### Synthesis and characterization

**Scheme 1:**
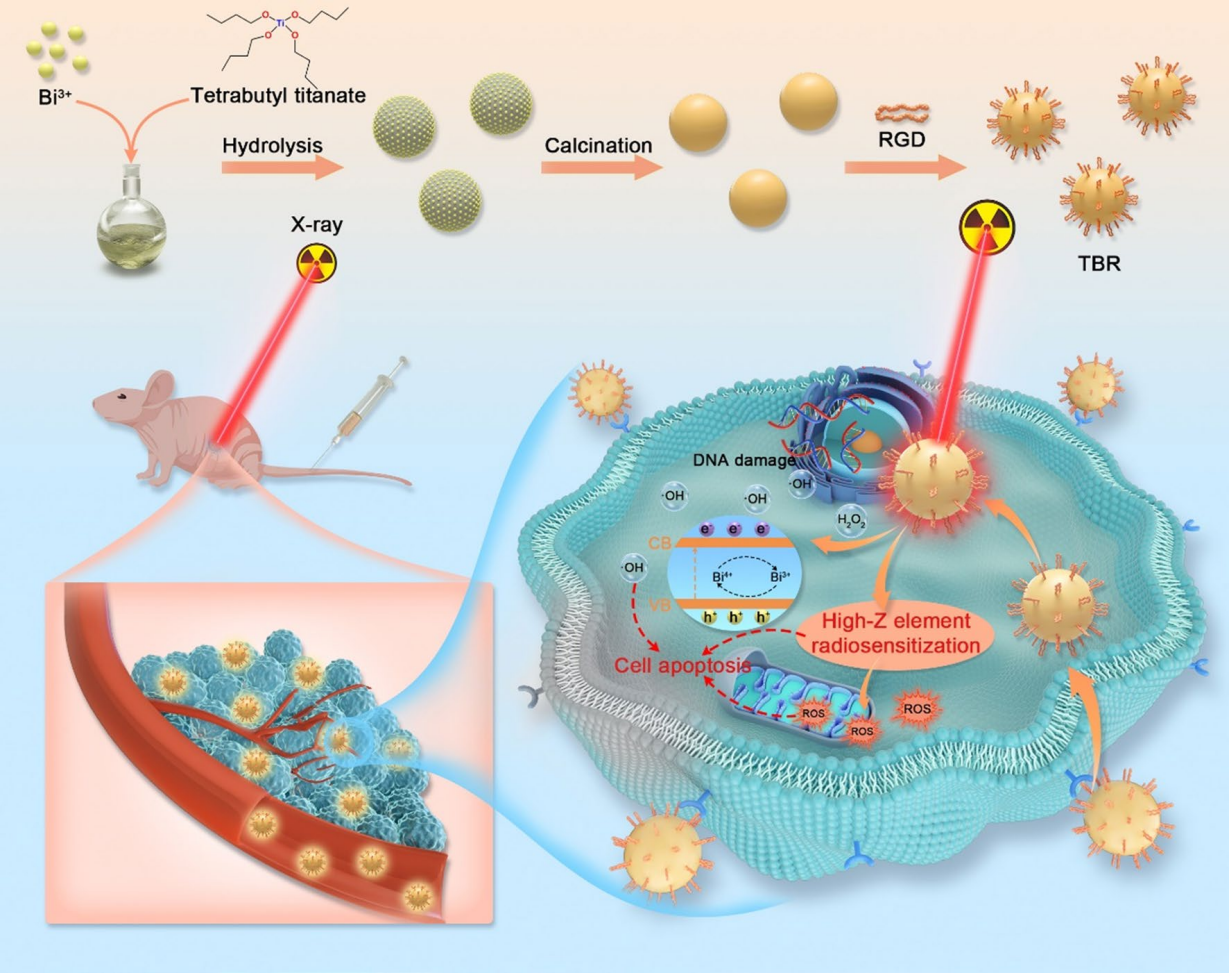
Schematic illustration of the preparation of TBR, and X-ray irradiated photodynamic therapy for U87MG tumors using TBR. TBR was intravenously injected and accumulated in U87MG tumors. Following X-ray activation, TBR efficiently promoted the electron–hole pair separation, producing large amount of cytotoxic ROS, which led to the nonreversible apoptosis of cancer cells

**Fig. 1:**
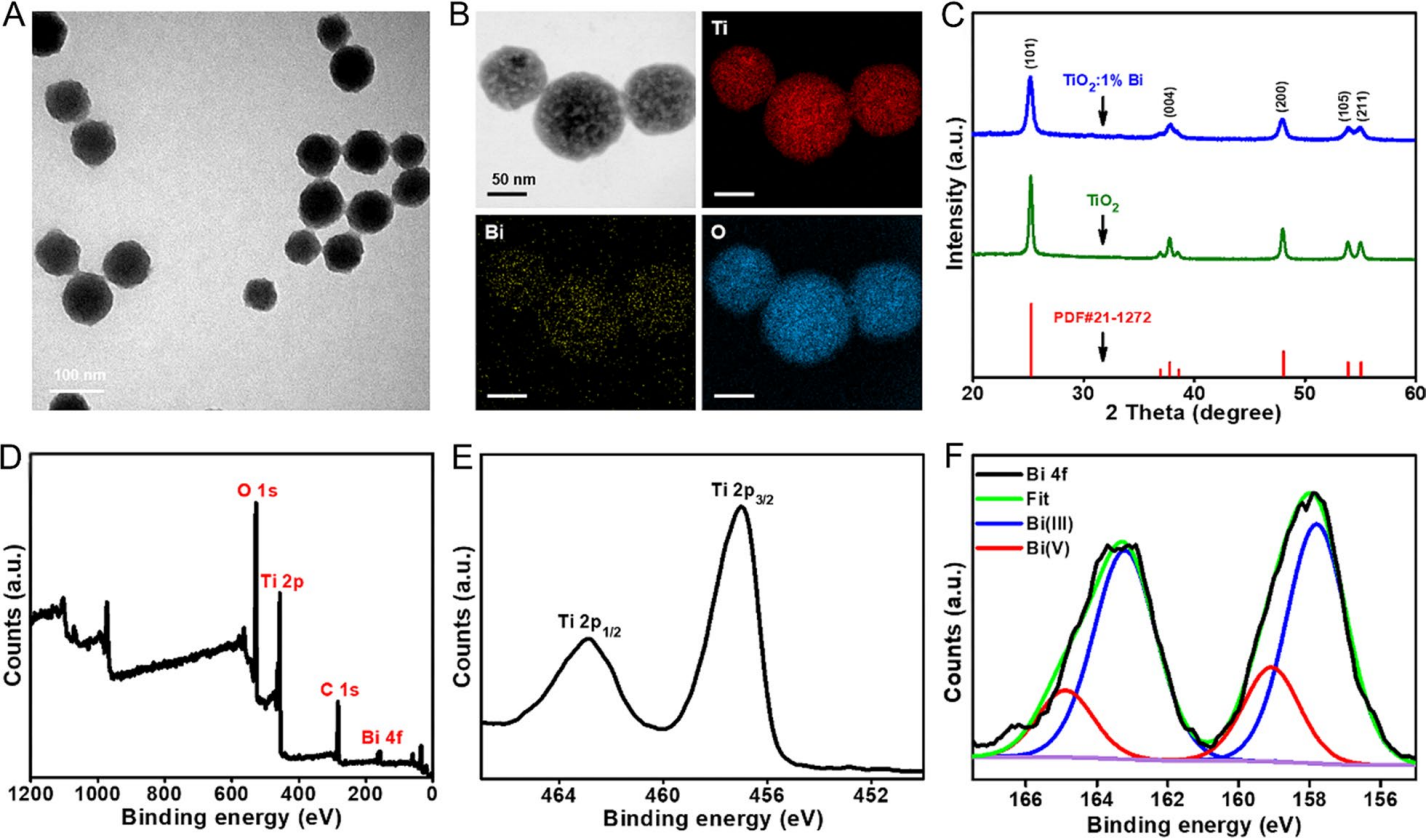
Characterization of TiO_2_:Bi (TB). **A** TEM image of TB. **B** HRTEM image of TB, and elemental distribution on TB. **C** X-ray diffraction patterns of TiO_2_ and TB. **D**–**F** XPS spectra of TB: **D** full spectrum, **E** Ti 2p spectrum, and **F** Bi 4f spectrum

Anatase TiO_2_ and TB nanoparticles (TB) were prepared by a sol–gel method (Scheme 1). The transmission electron microscopy (TEM) image revealed that the as-prepared TB was uniform spherical structures with a size of 78.6 ± 6.1 nm (Fig. 1A). High resolution TEM-mapping images revealed that Ti, O, and Bi elements evenly distributed in the nanoparticles (Fig. 1B). To further explore the influence of Bi-doping on the structure and properties of TiO_2_ matrix, we conducted the X-ray powder diffraction (XRD) analysis. As shown in Fig. 1C, the diffraction peaks of TB were completely consistent with the standard XRD pattern of anatase TiO_2_ (PDF #21-1272). The full-survey X-ray photoelectron spectra (XPS) of TB showed the characteristic peaks in the Ti, O, C, and Bi planes (Fig. 1D). The high-resolution XPS scanning of Ti 2p peaked at 457.0 eV (Ti 2p_3/2_) and 462.9 eV (Ti 2p_1/2_), and O 1 s peaked at 528.1 eV, further confirmed the existence of TiO_2_ in TB (Figs. 1E, Additional file 1: Fig. S1) [29]. The spectrum of Bi 4f showed two peaks at 157.8 eV and 163.3 eV, ascribed to Bi 4f_7/2_ and Bi 4f_5/2_, respectively. The Bi 4f spectrum can be deconvoluted into Bi(III) and Bi(V), which indicated that partial oxidation of Bi(III) to Bi(V). Bi(IV) may be a transition state in the synthesis of TB (Fig. 1F) [30, 31]. We speculated that Bi replaced the position of Ti in the anatase TiO_2_ and increase oxygen vacancies, as a result, photocatalytic activity will be enhanced.

### X-ray induced ROS-generation

**Fig. 2:**
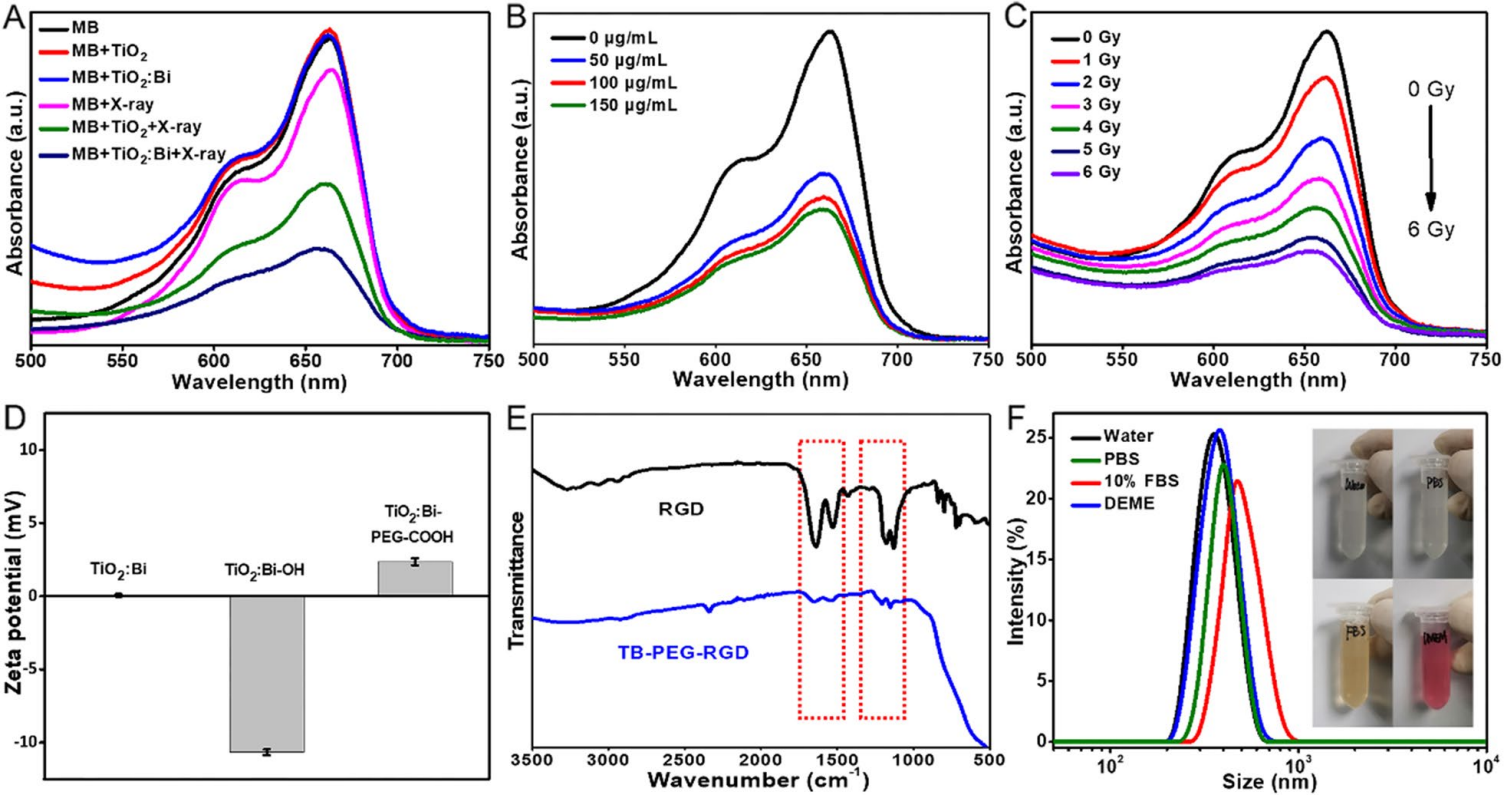
ROS production of TB and characterization of TBR. **A** The absorption spectra of MB with different treatments. **B** The absorption spectra of MB in different concentration of TB solution. **C** The absorption spectra of MB in TB solution under different dosage of X-ray irradiation. **D** Zeta potentials of TiO_2_:Bi, TiO_2_:Bi-OH, and TiO_2_:Bi-PEG-COOH. **E** FTIR spectra of RGD and TiO_2_:Bi-RGD (TBR). **F** The particle size distribution and colloid stability of TBR in different media, including water, PBS, 10%FBS, and DMEM

Anatase TiO_2_, due to its wide band gap and more oxygen vacancies, has strong ability to capture electrons. The doping of high-Z elements could further increase the hole-electron separation and promote the active oxygen species production [32, 33]. To explore the photosensitive catalytic activity of TB, we firstly studied the influence of Bi-doping amount on the crystal type of TiO_2_ matrix by comparing the XRD patterns of TB with different Bi content. The XRD results revealed that lower doped ratio (< 5%) keep the crystal structures of anatase TiO_2_. With the increase of Bi content, the characteristic diffraction peak of anatase TiO_2_ matrix gradually weakened. When it reached 10%, the diffraction peak disappeared obviously, which was consistent with the uncalcined morphology, and the anatase structure could no longer be maintained (Additional file 1: Fig. S2). Then, methylene blue (MB) was used as detection probe for hydroxyl radicals, to explore the photochemical properties of TB with different Bi-doping amounts under X-ray irradiation. MB contains nitrogen and sulfur chromophores with lone pair electrons attached to the benzene ring, which can react with hydroxyl radicals to generate hydroxylated MB, thus the color of solution changes from blue to colorless [26, 34]. By comparison, TB with Bi doping content of 1% showed the best MB-degradation performance, i.e., the best photosensitive activity, and the characteristic absorption peak of MB molecule was weakened obviously (Additional file 1: Fig. S3). Next, we compared the MB degradation abilities of undoped anatase TiO_2_ nanoparticles and TB (1% Bi-doping) with or without X-ray excitation. As shown in Fig. 2A, the 1% Bi-dopant significantly enhanced the ROS-production of anatase TiO_2_ nanoparticles under the same dose of X-ray irradiation. Meanwhile, the photosensitive catalysis activity of TB was positively correlated with the TB concentration and X-rays dose (Fig. 2B, C).

Therefore, TB with Bi doping content of 1% was applied for the subsequent biological evaluation. First, PEG was linked on TB for improving the biocompatibility. The change of surface zeta potential from − 10.67 mV (TiO_2_:Bi-OH) to 2.63 mV (TiO_2_:Bi-PEG-COOH) confirmed the successful PEGylation (Fig. 2D). The cyclopeptide RGD, which targets the integrin αvβ3 receptor on U87MG cells, was then coupled to the TB for getting active U87MG cell uptakes. As shown in Fig. 2E, the Fourier transform infrared spectroscopy (FTIR) of TiO_2_:Bi-PEG-RGD (TBR) nanoplatforms showed main characteristic peaks of RGD, manifesting the successful modification. Moreover, this novel TBR nanoplatform showed great colloidal stability and biosafety in various media (Fig. 2F). These results lay the foundation for its biological application.

### In vitro evaluations

**Fig. 3:**
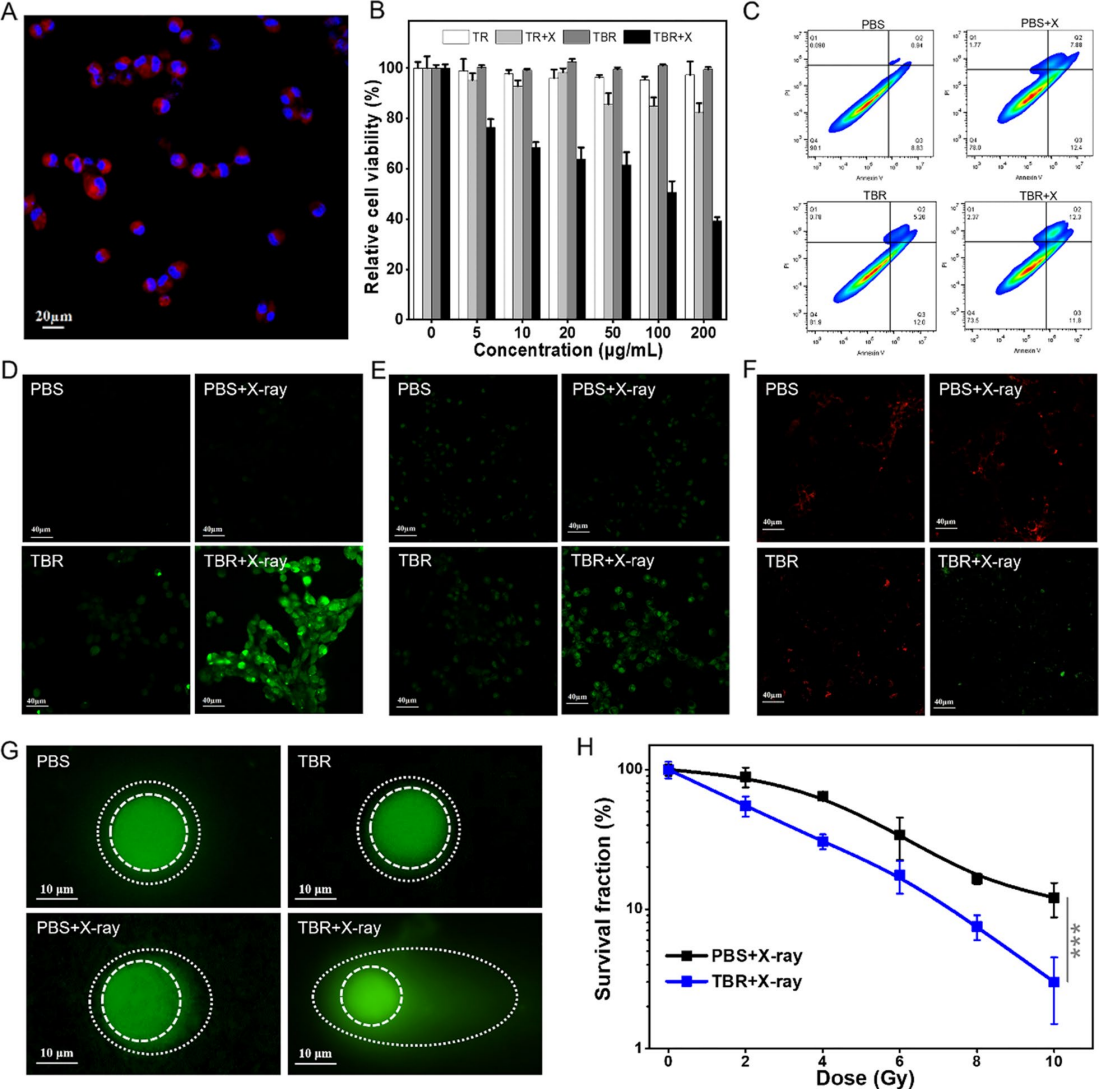
TBR-mediated radiosensitization of cancer cells. **A** Confocal image of the U87MG cells after incubating with TBR-Cy5.5 for 24 h. Blue and red colors represented Hoechst 33,342 and Cy5.5 fluorescence, respectively. **B** The viability of U87MG cells treated with 4 Gy of X-ray irradiation or without X-ray irradiation, after incubation with different concentrations of TBR. **C** Flow cytometric analysis of U87MG cells after various treatments. **D** Fluorescent images of U87MG cells stained by DCFH-DA after different treatments. Green fluorescence indicated the presence of ∙OH. **E** Fluorescent images of U87MG cells in BODIPY-C11 staining assay. **F** Fluorescent images of U87MG cells in JC-1 staining assay. **G** Comet assay of U87MG cells after treatment with PBS, PBS + X, TBR or TBR + X (X: X‐ray irradiation, 4 Gy). **H** Clonogenic cell survival assay

The anti-cancer efficiency of TBR was evaluated using malignant glioma U87MG cells. First, the fluorescent dye Cy5.5 (Ex/Em: 675/707 nm) was labeled to the TBR to visualize its uptakes in U87MG cells. As shown in Fig. 3A and Additional file 1: Fig. S4, obvious red fluorescence signals were mainly observed in U87MG cytoplasm after incubation with TBR-Cy5.5 for 12 h, confirming the effective internalization of TBR into U87MG cells by receptor-mediated endocytosis. The TBR showed no obvious cytotoxicity to normal mouse fibroblasts (Additional file 1: Fig. S5). Moreover, we compared the in vitro X-PDT effects of TiO_2_ and TB. Satisfyingly, TBs had a more obvious killing effect on U87MG cells after irradiation of X-rays (4 Gy) with 60.8 ± 1.6% cell lethality (Fig. 3B). Further, we employed flow cytometry to quantitatively evaluate the ability of photosensitive TBR to induce apoptosis (Fig. 3C, Additional file 1: Fig. S6). In contrast to the PBS, TBR, and PBS + X-ray groups, the proportion of living cells (showed in Fig. 3C, Q4) in TBR + X-ray treatment group decreased from 90.1 to 73.5%. And the cells in the stage of late apoptosis were also increasing to 12.3%. It is proved that TBR nanoplatform can induce significant necrosis and apoptosis of U87MG cells under X-ray irradiation. Compared with the PBS group, the apoptosis rate (Additional file 1: Fig. S6) of the TBR + X group increased from 10.2 to 26.4%, performing the potential ability to damage the U87MG cells.

The mechanism of cancer cell killing was then investigated through measuring the biological behavior changes of U87MG cells in a systematic way. Firstly, DCFH-DA was used to detect the intracellular hydroxyl radicals, which emit green fluorescence after reaction with ROS. Under the same dosage of X-rays activation, U87MG cells treated with photosensitive TBR exhibited stronger green fluorescence signals than the control groups, indicating more ROS generation (Fig. 3D, Additional file 1: Fig. S7). Large amount of cytotoxic ROS induced high lipid peroxidation level, *i.e.* photodynamic therapy effect, which was tested through BODIPY-C11 staining assay (Fig. 3E, Additional file 1: Fig. S8). TBR + X-ray treatment group has stronger green fluorescence (same cells number in different group), which means higher lipid peroxidation. It shows that more ROS had been produced to cause cell membrane damage in TBR + X-ray group [35]. Furthermore, the mitochondria membrane potential of U87MG cells was determined by JC-1 staining assay. Quantitative analysis mitochondrial membrane potential (MMP) changes also reveal the function of mitochondrial. Compared with the control groups, the TBR + X-rays group showed weaker red fluorescence and stronger green fluorescence, causing severe mitochondria dysfunction The sharply drop of MMP indicated cell apoptosis (Fig. 3F, Additional file 1: Fig. S9). The nuclear damage was then measured. As the results of single-cell electrophoresis assay shown in Fig. 3G, Additional file 1: Fig. S10, TBR + X-rays treatment led to high frequency DNA strand breaks of U87MG cells, forming a comet-like smearing. This phenomenon demonstrated the great radiosensitizing effect of TBR nanoplatform [36]. Due to the enhanced radiotherapy effect, the U87MG cells treated with TBR + X-rays exhibited more proliferation decrease than that of X-rays alone, as determined using colony formation assay (Fig. 3H, Additional file 1: Table S1) [37]. The dose enhancement factor (DEF) was calculated to be 1.54. The above results suggested the obtained TBR could simultaneously achieve oxygen-independent type-I PDT effect and enhanced-radiotherapy effect under the low-dose of X-ray irradiation [38].

### In vivo evaluations

**Fig. 4:**
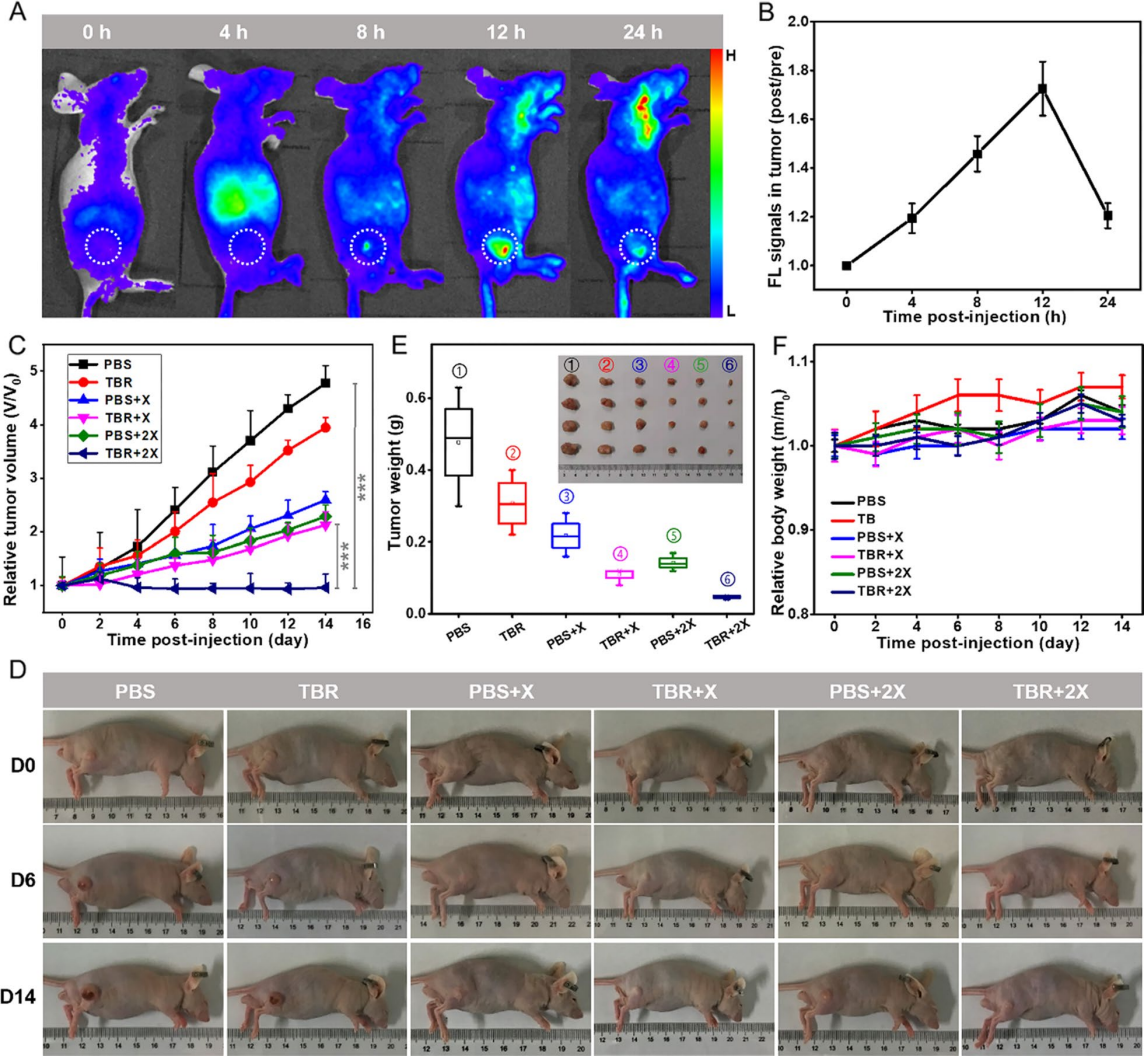
In vivo fluorescence images and cancer therapeutic efficacy of TBR. **A** Fluorescence imaging of mice bearing U87MG tumor at 0, 4, 8, 12, 24 h post-injection intravenously of TBR-Cy5.5. The dotted circles indicated subcutaneous tumors. **B** The corresponding fluorescence signals of subcutaneous tumors at 0, 4, 8, 12, 24 h post-injection intravenously of TBR-Cy5.5. **C** Tumor volume curves in 14 days after different treatments. **D** Typical photographs of mice bearing U87MG tumor with different treatments at day 0, 6, and 14. **E** U87MG tumor photographs and weights at the end of treatments. **F** Body weight curves of mice with different treatments

To accomplish the in vivo application, we firstly evaluated the long-term biosafety in vivo through blood biochemistry and hematology analysis at day 3 and day 7 post-intravenous injection of TBR in BALB/c nude mice. The main blood biochemical indices and blood routine examination items of the experimental mice were within normal range, and there was no significant difference with the control group (day 0) (Additional file 1: Figs. S11, S12; Tables S2, S3). Based on the good biocompatibility of TBR, we further investigated the therapeutic efficacy for U87MG tumors in vivo. The subcutaneous U87MG BALB/c xenograft tumor models were established. The in vivo fluorescence imaging of U87MG tumor-bearing mice was carried out at 0, 4, 8, 12, and 24 h after intravenous injection of Cy5.5-TBR. As shown in Fig. 4A, B, the TBR could successfully target and accumulate in tumor tissues via the combined active receptor-binding and passive enhanced permeability and retention (EPR) processes, exhibiting the highest uptakes in tumors at 12 h. After, the mice bearing U87MG tumors were randomly divided into six groups (n = 5), namely PBS, PBS + X, PBS + 2X, TBR, TBR + X, and TBR + 2X (X, X-ray irradiation) groups, and 4 Gy of X-ray treatment was applied to tumor tissue at 12 h after intravenous injection of PBS or TBR. The anti-tumor effect was observed and tumor growth inhibitory rate was calculated. The TBR + 2X treatment could significantly suppress U87MG tumor growth and prolong the survival of tumor-bearing mice, in contrast to control groups (Fig. 4C, D). The tumor inhibition rate was 79.8% (compared to PBS group), indicating the great therapeutic effect of TBR mediated X-PDT (Fig. 4E). Moreover, the experimental mice maintained their weight, and had no behavioral abnormalities (Fig. 4F). The H&E staining analysis showed no pathological change in the major organs and tissues of mice (Additional file 1: Fig. S13). The TBR effectively concentrated radiation energy on the tumor area, destroying tumor cells without damaging normal tissue.

## Conclusion

In summary, composition tunability of semiconductor radiosensitizers are present for low-dose X-ray induced photodynamic therapy. The doping of high-Z element Bi into the lattice of anatase TiO_2_ increased X-ray absorption, boosting the generation of ROS. Only 1% of doping achieved dramatical ROS-generation under low-dose (4 Gy) irradiation. Investigations in vitro and in vivo indicated that the semiconductor radiosensitizers are biocompatible; more importantly, under low-dose X-ray irradiation, our nanosensitizers produced effectively ROS in cancer cells, causing significant mitochondria damages, membrane lipid peroxidation, and nuclei destruction. The synergistic therapy and radiosensitization greatly destroyed cancer cells without harming healthy cells nearby. The present work suggests a promising strategy for the design and tunability of semiconductor radiosensitizers for cancer management.

## Supplementary Information

**Additional file 1: Figure S1.** XPS spectrum of O 1 s. **Figure S2.** X-ray diffraction patterns of TiO_2_:Bi (TB) with different contents of Bi dopant. **Figure S3.** The absorption spectra of MB treated with TB (Bi doping amount: 1%, 2%, 5%, and 10%) under X-ray irradiation. **Figure S4.** Confocal images of the U87MG cells after incubating with TBR-Cy5.5 for 24 h. Blue and red colors represented Hoechst 33342 and Cy5.5 fluorescence. **Figure S5.** The viability of mouse fibroblasts treated with different concentrations of TBR. **Figure S6.** Cell apoptosis determined using Annexin V-FITC/Propidium Iodide apoptosis assay (**P < 0.05). **Figure S7.** CLSM evaluation of U87MG cells stained by DCFH-DA after different treatments. Green fluorescence indicated the presence of OH. **Figure S8. A.** CLSM evaluation of U87MG cells in BODIPY-C11 staining assay..B. lipoperoxides, based on BODIPY staining results (**P < 0.05). **Figure S9. A.** CLSM observation of U87MG cells in JC-1 staining assay. The red fluorescence indicates that the membrane potential is positive, and the green fluorescence indicates that the membrane potential decreases. B. The membrane potential (ΔΨm) changes, assessed by JC-1staining. **Figure S10.** Lower magnification images with multiple cells of comet assay. **Figure S11.** Mice were intravenously treated daily for 3 days with TBR (20 mg/kg). Blood samples were collected for serum chemistry analysis before treatment (day 0), and at day 3 and day 7 post- intravenous treatment. AST, aspartate transaminase; ALT, alanine transaminase; UREA, blood urea nitrogen; CREA, creatinine. **Figure S12.** Mice were intravenously treated daily for 3 days with PBS or TBR (20 mg/kg). Blood samples were collected for complete blood analysis before treatment (day 0), and at day 3 and day 7 post- intravenous treatment. WBC, white blood cell; RBC, red blood cell; HGB, hemoglobin; HCT, hematocrit; MCV, mean corpuscular volume; MCH, mean corpuscular hemoglobin; MCHC, mean corpuscular hemoglobin concentration; RDW-SD, RBC distribution width; PLT, platelets; MPV, mean platelet volume. **Figure S13.** H&E staining of main organs of mice after different treatments (Scale bar: 100 µm). **Table S1.** Radiation enhancement related factor values of TBR by clonogenic assay. **Table S2**. Serum chemistry of mice after intravenous injection with TBR. Data are mean ± s.d. **Table S3.** Complete blood count of mice after intravenous injection with TBR. Data are mean ± s.d.

## Data Availability

The datasets used and analyzed during the current study are available from the corresponding author on reasonable request.

## References

[1] Weichselbaum RR, Liang H, Deng L, Fu YX (2017). Radiotherapy and immunotherapy: a beneficial liaison?. Nat Rev Clin Oncol.

[2] Song G, Cheng L, Chao Y, Yang K, Liu Z (2017). Emerging nanotechnology and advanced materials for cancer radiation therapy. Adv Mater.

[3] Chen X, Song J, Chen X, Yang H (2019). X-ray-activated nanosystems for theranostic applications. Chem Soc Rev.

[4] Chong LM, Tng DJH, Tan LLY, Chua MLK, Zhang Y (2021). Recent advances in radiation therapy and photodynamic therapy. Appl Phys Rev.

[5] Chen H, Wang GD, Chuang YJ, Zhen Z, Chen X, Biddinger P, Hao Z, Liu F, Shen B, Pan Z (2015). Nanoscintillator-mediated X-ray inducible photodynamic therapy for in vivo cancer treatment. Nano Lett.

[6] Sun W, Luo L, Feng Y, Cai Y, Zhuang Y, Xie RJ, Chen X, Chen H (2020). Aggregation-induced emission gold clustoluminogens for enhanced low-dose X-ray-enduced photodynamic therapy. Angew Chem Int Ed.

[7] Fan W, Tang W, Lau J, Shen Z, Xie J, Shi J, Chen X (2019). Breaking the depth dependence by nanotechnology-enhanced X-ray-excited deep cancer theranostics. Adv Mater.

[8] Sun W, Zhou Z, Pratx G, Chen X, Chen H (2020). Nanoscintillator-mediated X-ray induced photodynamic therapy for deep-seated tumors: from concept to biomedical applications. Theranostics.

[9] Hu J, Tang Y, Elmenoufy AH, Xu H, Cheng Z, Yang X (2015). Nanocomposite-based photodynamic therapy strategies for deep tumor treatment. Small.

[10] Wang Y-Y, Liu Y-C, Sun H, Guo D-S (2019). Type I photodynamic therapy by organic–inorganic hybrid materials: from strategies to applications. Coordin Chem Rev.

[11] Liu JN, Bu W, Shi J (2017). Chemical design and synthesis of functionalized probes for imaging and treating tumor hypoxia. Chem Rev.

[12] Zhou Z, Song J, Nie L, Chen X (2016). Reactive oxygen species generating systems meeting challenges of photodynamic cancer therapy. Chem Soc Rev.

[13] Xie J, Gong L, Zhu S, Yong Y, Gu Z, Zhao Y (2019). Emerging strategies of nanomaterial-mediated tumor radiosensitization. Adv Mater.

[14] Jing X, Yang F, Shao C, Wei K, Xie M, Shen H, Shu Y (2019). Role of hypoxia in cancer therapy by regulating the tumor microenvironment. Mol Cancer.

[15] Chen Y, Li N, Wang J, Zhang X, Pan W, Yu L, Tang B (2019). Enhancement of mitochondrial ROS accumulation and radiotherapeutic efficacy using a Gd-doped titania nanosensitizer. Theranostics.

[16] Clement S, Campbell JM, Deng W, Guller A, Nisar S, Liu G, Wilson BC, Goldys EM (2020). Mechanisms for tuning engineered nanomaterials to enhance radiation therapy of cancer. Adv Sci.

[17] Zhang C, Zhao K, Bu W, Ni D, Liu Y, Feng J, Shi J (2015). Marriage of scintillator and semiconductor for synchronous radiotherapy and deep photodynamic therapy with diminished oxygen dependence. Angew Chem Int Ed.

[18] Nakayama M, Sasaki R, Ogino C, Tanaka T, Morita K, Umetsu M, Ohara S, Tan Z, Nishimura Y, Akasaka H (2016). Titanium peroxide nanoparticles enhanced cytotoxic effects of X-ray irradiation against pancreatic cancer model through reactive oxygen species generation in vitro and in vivo. Radiat Oncol.

[19] Zheng L, Zhu R, Chen L, Fu Q, Li J, Chen C, Song J, Yang H (2021). X-ray sensitive high-Z metal nanocrystals for cancer imaging and therapy. Nano Res.

[20] Chen Q, Chen J, Yang Z, Xu J, Xu L, Liang C, Han X, Liu Z (2019). Nanoparticle-enhanced radiotherapy to trigger robust cancer immunotherapy. Adv Mater.

[21] Anselmo AC, Mitragotri S (2019). Nanoparticles in the clinic: an update. Bioeng Transl Med.

[22] Li J, Xie L, Sang W, Li W, Wang G, Yan J, Zhang Z, Tian H, Fan Q, Dai Y (2022). A metal-phenolic nanosensitizer performs hydrogen sulfide-reprogrammed oxygen metabolism for cancer radiotherapy intensification and immunogenicity. Angew Chem Int Ed.

[23] Rabin O, Manuel Perez J, Grimm J, Wojtkiewicz G, Weissleder R (2006). An X-ray computed tomography imaging agent based on long-circulating bismuth sulphide nanoparticles. Nat Mater.

[24] Liu T, Yang K, Liu Z (2020). Recent advances in functional nanomaterials for X-ray triggered cancer therapy. Progr Nat Sci Mate.

[25] Guo Z, Zhu S, Yong Y, Zhang X, Dong X, Du J, Xie J, Wang Q, Gu Z, Zhao Y (2017). Synthesis of BSA-coated BiOI@Bi_2_S_3_ semiconductor heterojunction nanoparticles and their applications for radio/photodynamic/photothermal synergistic therapy of tumor. Adv Mater.

[26] Gilson RC, Black KCL, Lane DD, Achilefu S (2017). Hybrid TiO_2_-ruthenium nano-photosensitizer synergistically produces reactive oxygen species in both hypoxic and normoxic conditions. Angew Chem Int Ed.

[27] Cheng K, Sano M, Jenkins CH, Zhang G, Vernekohl D, Zhao W, Wei C, Zhang Y, Zhang Z, Liu Y (2018). Synergistically enhancing the therapeutic effect of radiation therapy with radiation activatable and reactive oxygen species-releasing nanostructures. ACS Nano.

[28] Wang XJ, Yang WY, Li FT, Zhao J, Liu RH, Liu SJ, Li B (2015). Construction of amorphous TiO_2_/BiOBr heterojunctions via facets coupling for enhanced photocatalytic activity. J Hazard Mater.

[29] Wang L, Cheng B, Zhang L, Yu J (2021). In situ irradiated XPS investigation on S-scheme TiO_2_ @ZnIn_2_S_4_ photocatalyst for efficient photocatalytic CO_2_ reduction. Small.

[30] Li H, Wang D, Wang P, Fan H, Xie T (2009). Synthesis and studies of the visible-light photocatalytic properties of near-monodisperse bi-doped TiO_2_ nanospheres. Chemistry.

[31] Barreca D, Morazzoni F, Andrea Rizzi G, Scotti R, Tondello E (2001). Molecular oxygen interaction with Bi_2_O_3_: a spectroscopic and spectromagnetic investigation. Phys Chem Chem Phys.

[32] Wang B, Biesold GM, Zhang M, Lin Z (2021). Amorphous inorganic semiconductors for the development of solar cell, photoelectrocatalytic and photocatalytic applications. Chem Soc Rev.

[33] Chen H, Li J, Yang W, Balaghi SE, Triana CA, Mavrokefalos CK, Patzke GR (2021). The role of surface states on reduced TiO_2_@BiVO_4_ photoanodes: enhanced water oxidation performance through improved charge transfer. ACS Catal.

[34] Shi Z, Meng X, Zhang K, Tang S, Zhang C, Yang Z, Dong H, Zhang X (2021). Engineering structural metal–organic framework for hypoxia-tolerant type I photodynamic therapy against hypoxic cancer. ACS Mater Lett.

[35] Hassannia B, Vandenabeele P, Vanden Berghe T (2019). Targeting ferroptosis to iron out cancer. Cancer Cell.

[36] Sun W, Luo L, Feng Y, Qiu Y, Shi C, Meng S, Chen X, Chen H (2020). Gadolinium–rose bengal coordination polymer nanodots for MR-/fluorescence-image-guided radiation and photodynamic therapy. Adv Mater.

[37] Guo X, Yang N, Ji W, Zhang H, Dong X, Zhou Z, Li L, Shen HM, Yao SQ, Huang W (2021). Mito-bomb: targeting mitochondria for cancer therapy. Adv Mater.

[38] Lan G, Ni K, Veroneau SS, Feng X, Nash GT, Luo T, Xu Z, Lin W (2019). Titanium-based nanoscale metal-organic framework for type I photodynamic therapy. J Am Chem Soc.

