# Supplementary material for: Composition tunability of semiconductor radiosensitizers for low-dose X-ray induced photodynamic therapy
Source: J Nanobiotechnology. 2022 Jun 21;20:293. doi: 10.1186/s12951-022-01494-7 (PMC9210653; doi:10.1186/s12951-022-01494-7)
Supplement: Supplementary file 1 — Additional file 1: Figure S1. XPS spectrum of O 1 s. Figure S2. X-ray diffraction patterns of TiO2:Bi (TB) with different contents of Bi dopant. Figure S3. The absorption spectra of MB treated with TB (Bi doping amount: 1%, 2%, 5%, and 10%) under X-ray irradiation. Figure S4. Confocal images of the U87MG cells after incubating with TBR-Cy5.5 for 24 h. Blue and red colors represented Hoechst 33342 and Cy5.5 fluorescence. Figure S5. The viability of mouse fibroblasts treated with different concentrations of TBR. Figure S6. Cell apoptosis determined using Annexin V-FITC/Propidium Iodide apoptosis assay (**P < 0.05). Figure S7. CLSM evaluation of U87MG cells stained by DCFH-DA after different treatments. Green fluorescence indicated the presence of OH. Figure S8. A. CLSM evaluation of U87MG cells in BODIPY-C11 staining assay..B. lipoperoxides, based on BODIPY staining results (**P < 0.05). Figure S9. A. CLSM observation of U87MG cells in JC-1 staining assay. The red fluorescence indicates that the membrane potential is positive, and the green fluorescence indicates that the membrane potential decreases. B. The membrane potential (ΔΨm) changes, assessed by JC-1staining. Figure S10. Lower magnification images with multiple cells of comet assay. Figure S11. Mice were intravenously treated daily for 3 days with TBR (20 mg/kg). Blood samples were collected for serum chemistry analysis before treatment (day 0), and at day 3 and day 7 post- intravenous treatment. AST, aspartate transaminase; ALT, alanine transaminase; UREA, blood urea nitrogen; CREA, creatinine. Figure S12. Mice were intravenously treated daily for 3 days with PBS or TBR (20 mg/kg). Blood samples were collected for complete blood analysis before treatment (day 0), and at day 3 and day 7 post- intravenous treatment. WBC, white blood cell; RBC, red blood cell; HGB, hemoglobin; HCT, hematocrit; MCV, mean corpuscular volume; MCH, mean corpuscular hemoglobin; MCHC, mean corpuscular hemoglobin concentration; RDW-SD, [file 12951_2022_1494_MOESM1_ESM.docx]

**Additional file 1**

**Composition tunability of semiconductor radiosensitizers for low-dose X-ray induced photodynamic therapy**

Lei Chen^1^, Jinghui Zhang^1,2^, Lihua Xu^1^, Luchao Zhu^4^, Jinpeng Jing^1^, Yushuo Feng^1^, Zongzhang Wang^1^, Peifei Liu^1^, Wenjing Sun*^1,3^, Xiangmei Liu*^2^, Yimin Li*^4,5^, Hongmin Chen*^1,2,4^

^1^State Key Laboratory of Molecular Vaccinology and Molecular Diagnostics & Center for Molecular Imaging and Translational Medicine, School of Public Health, Xiamen University, Xiamen 361102, P. R. China

E-mail: hchen@xmu.edu.cn

^2^State Key Laboratory of Organic Electronics and Information Displays & Institute of Advanced Materials (IAM), Jiangsu Key Laboratory for Biosensors, Nanjing University of Posts & Telecommunications, Nanjing 210023, P. R. China

E-mail: iamxmliu@njupt.edu.cn

^3^ZJU-Hangzhou Global Scientific and Technological Innovation Center, Hangzhou 311200, P. R. China

E-mail: sunwj0102@zju.edu.cn

^4^Department of Radiation Oncology, Cancer Center, the First Affiliated Hospital of Xiamen University, Xiamen 361003, P. R. China

E-mail: lym05@xmu.edu.cn

^5^The Third Clinical Medical College, Fujian Medical University, P. R. China

**Experimental Section:**

**Materials**

Bismuth nitrate pentahydrate (Bi(NO_3_)_3_·5H_2_O), tetrabutyl titanate (TBOT), 4-nitrophenyl chloroformate 3-(4,5-dimethylthiazol-2-yl)-2,5-diphenyltetrazolium bromide (MTT) and 2,7-dichlorodihydrofluorescein diacetate (DCFH-DA) were purchased from Sigma-Aldrich (MO, USA). Methylene blue (MB) was purchased from Alfa Aesar (China) Chemical Co. Ltd. Hoechst 33342 and BODIPY 581/591 C11 were purchased from Thermo Fisher Scientific (Waltham, MA). JC-1 was purchased from MedChemExpress (MCE). U87MG cells were obtained from Institute of Biochemistry and Cell Biology (Shanghai, China). Male nude mice were purchased from Shanghai SLAC Laboratory Animal Co. Ltd (Shanghai, China).

**Characterization**

TEM images were taken on a JEM-2100 transmission electron microscope (JEOL Ltd., Tokyo, Japan). HRTEM images and element mapping were taken on a Tecnai F30 transmission electron microscope. The crystalline phase was examined by X-ray diffraction (XRD) at 40 kV and 30 mA in the range of 20–60° (2θ) at a scanning rate of 5°/min. The hydrodynamic diameters and [zeta potential](http://webvpn.xmu.edu.cn/https/77726476706e69737468656265737421e7e056d234336155700b8ca891472636a6d29e640e/topics/chemistry/zeta-potential) of [nanoparticles](http://webvpn.xmu.edu.cn/https/77726476706e69737468656265737421e7e056d234336155700b8ca891472636a6d29e640e/topics/chemistry/nanoparticle) were measured on Malvern Zetasizer nano-ZS90. FTIR spectra were obtained using Bruker Vertex 70V spectrometer, scanning from 3500 to 500 cm^−1^, and the samples were prepared by the KBr pellet method. XPS was performed using a PHI 5000 Versa Probe with Al Kα as the excitation source. The MTT assay of the TBR was measured by Enzyme-labelled meter: BioTek CO., Ltd (USA). The fluorescence images of cells were taken on a laser scanning confocal microscopy (Olympus FV1200, Japan). The whole-body fluorescence imaging of mice was conducted using an IVIS in vivo imaging system (Caliper IVIS, Lumina II).

**Cell culture**

U87MG (human glioblastoma) cell lines and 3T3 cell lines (mouse embryo fibroblasts) were cultured at 37 ^o^C within 5% CO_2_.

**Animal experiments**

All the animal experimental procedures were in accord with the guidelines of the Regional Ethics Committee for Animal Experiments and the Care Regulations approved by the Institutional Animal Care and Use Committee of Xiamen University. Mice bearing U87MG tumors were constructed by subcutaneously injecting 2×10^6^ U87MG cells into the right hind leg of male BALB/c nude mice. When the tumor reached to 50-80 mm^3^ in volume, the BALB/c mice bearing U87MG tumors were applied. The mice bearing U87MG tumors were randomly divided into 6 groups (n=5), namely PBS, PBS+X, PBS+2X, TBR, TBR+X, and TBR+2X (X, X-ray irradiation) groups, and X-ray treatment was applied to the tumor region after intravenous injection of PBS or TBR (5 mg/kg). Group PBS+2X and TBR+2X repeated the therapeutic process on the third day.

**In vivo imaging evaluation**

Mice bearing U87MG tumors were intravenously injected with TBR-Cy5.5 (5 mg/kg). Fluorescence imaging was conducted at 0, 4, 8, 12, and 24 h post-injection.

**Serum chemistry and hematological analysis**

Mice were intravenously treated daily for 3 days with TBR (20 mg/kg). Blood samples were collected for serum chemistry analysis and complete blood analysis before treatment (day 0), and at day 3 and day 7 post-treatment. Aspartate aminotransferase (AST), alanine aminotransferase (ALT), urea nitrogen (UREA), and creatinine (CREA-S) in mice serum were measured using an Auto Biochemistry Analyzer (Mindray, BS-220). White blood cell (WBC), red blood cell (RBC), hemoglobin (HGB), hematocrit (HCT), mean corpuscular volume (MCV), mean corpuscular hemoglobin (MCH), mean corpuscular hemoglobin concentration (MCHC), RBC distribution width (RDW-SD), platelets (PLT), and mean platelet volume (MPV) in mice blood were measured using an Automatic Tri-classification Blood Cell Analyzer (Mindray, BC-2600).

**

**

**Figure S1.** O 1s spectrum of TB.

**
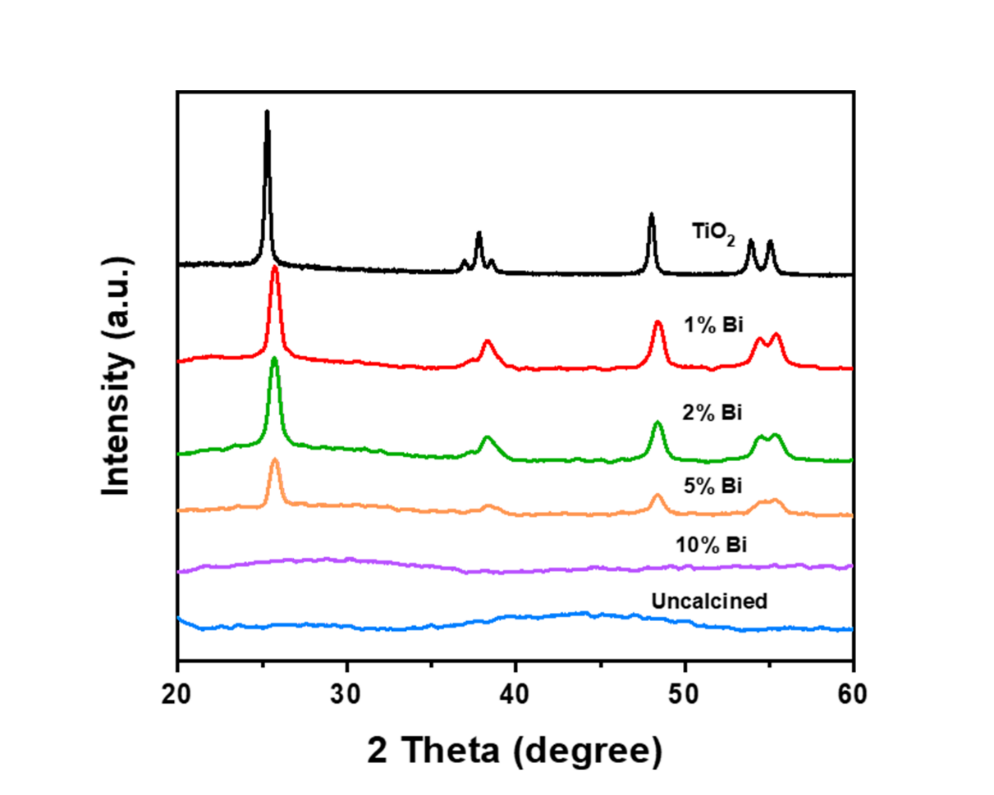
**

**Figure S2.** X-ray diffraction patterns of TiO_2_:Bi (TB) with different contents of Bi dopant.

**

**

**Figure S3.** The absorption spectra of MB (100 µg/mL) treated with TB (Bi doping amount: 1%, 2%, 5%, and 10%) under X-ray irradiation.

**
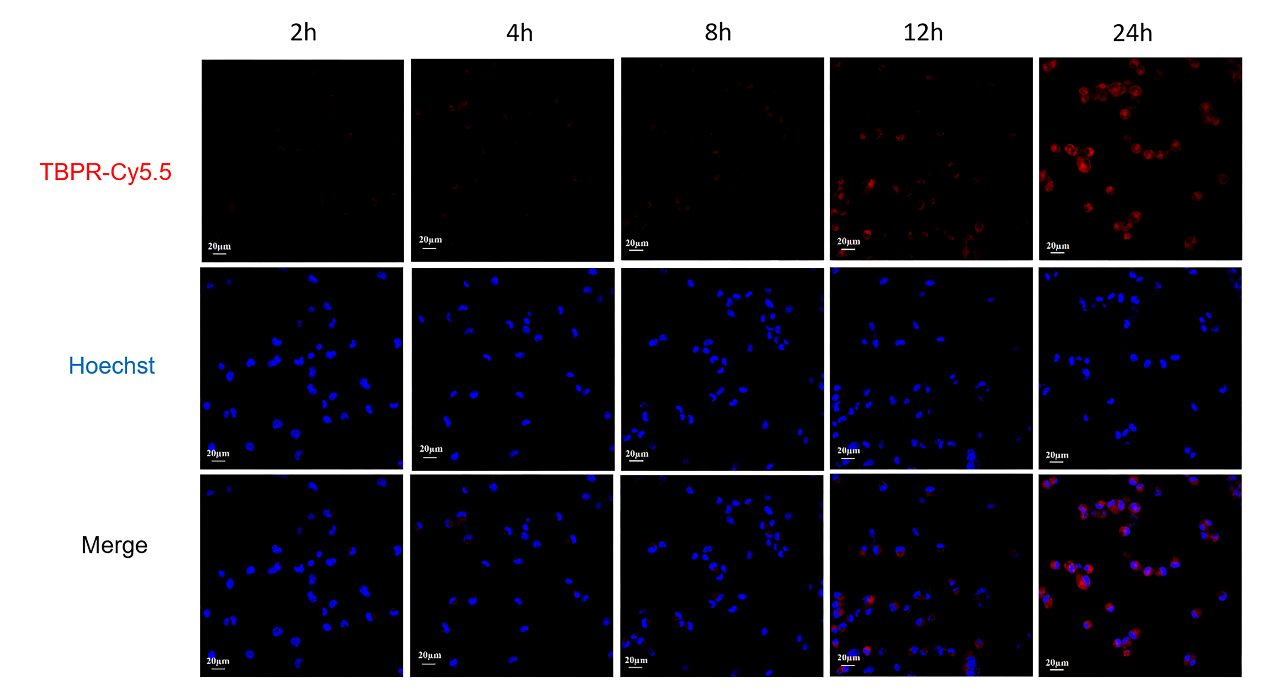
**

**Figure S4.** Confocal images of the U87MG cells after incubating with TBR-Cy5.5 for 24 h. Blue and red colors represented Hoechst 33342 and Cy5.5 fluorescence.

**
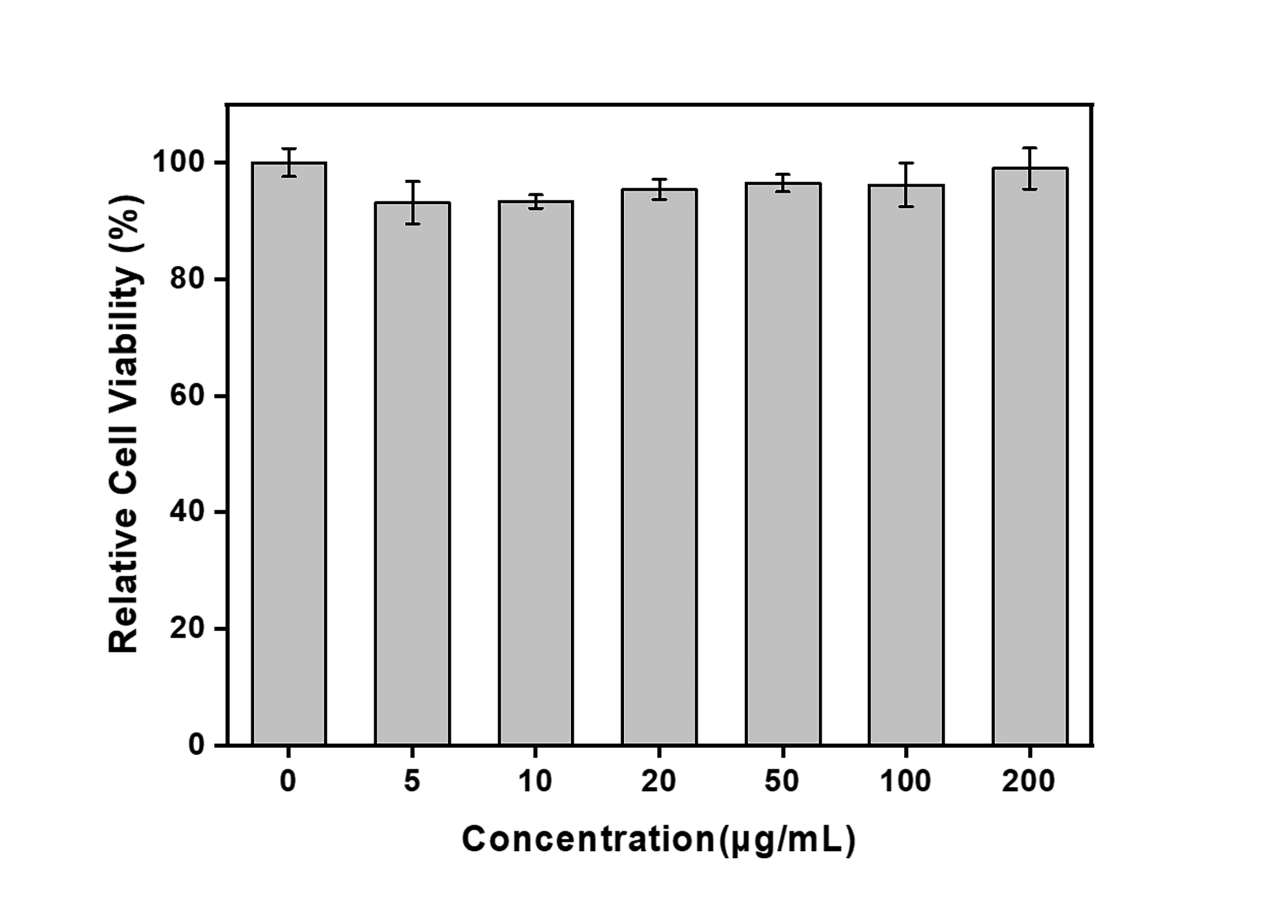
**

**Figure S5.** The viability of mouse fibroblasts treated with different concentrations of TBR (Six biologically independent samples were taken for each group).

**
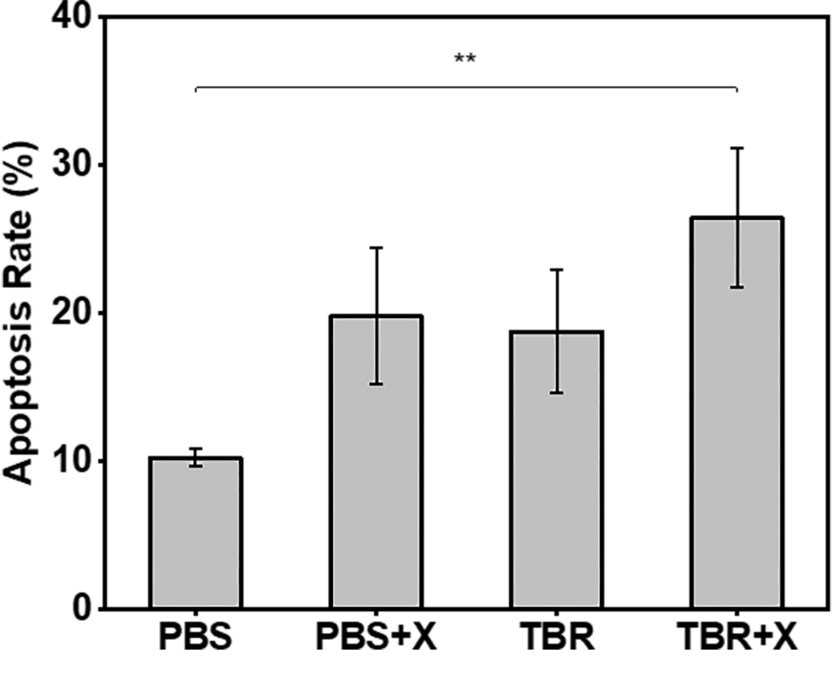
**

**Figure S6.** Cell apoptosis determined using Annexin V-FITC/Propidium Iodide apoptosis assay (**P < 0.05).

**
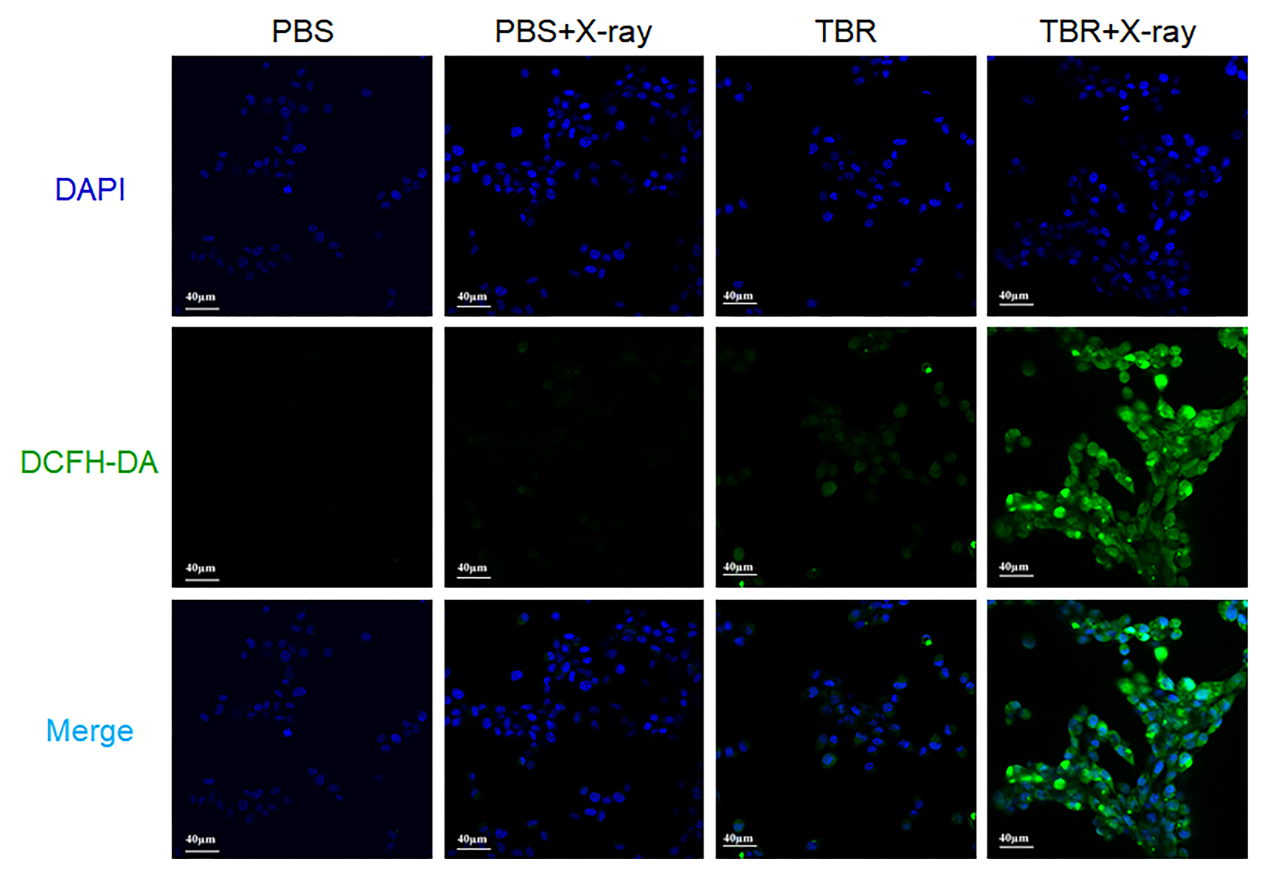
**

**Figure S7.** CLSM evaluation of U87MG cells stained by DCFH-DA after different treatments. Green fluorescence indicated the presence of 🞄OH.

**
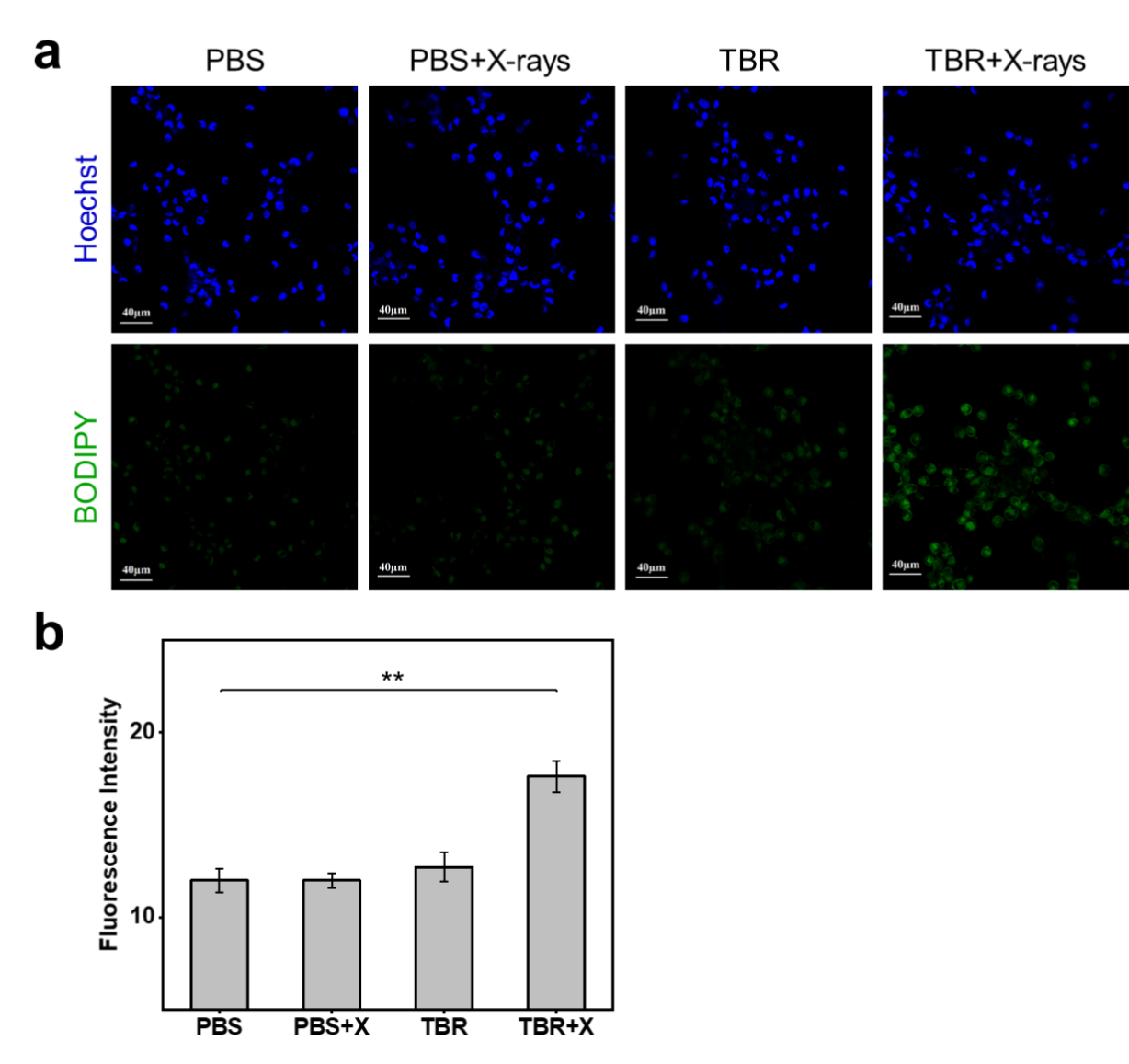
**

**Figure S8.** A. CLSM evaluation of U87MG cells in BODIPY-C11 staining assay. B. lipoperoxides, based on BODIPY staining results (**P < 0.05).


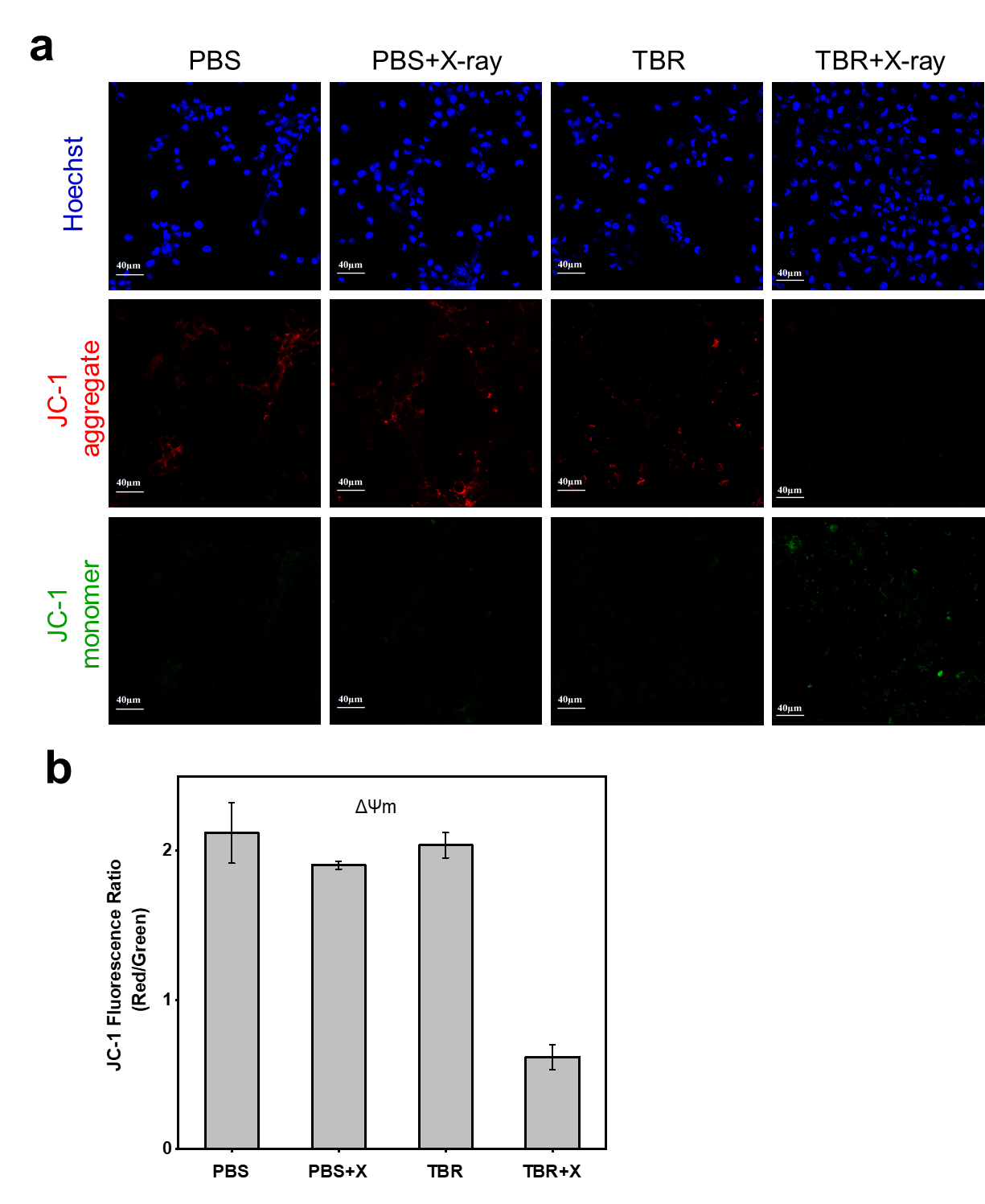


**Figure S9.** A. CLSM observation of U87MG cells in JC-1 staining assay. The red fluorescence indicates that the membrane potential is positive, and the green fluorescence indicates that the membrane potential decreases. B. The membrane potential (ΔΨm) changes, assessed by JC-1staining.


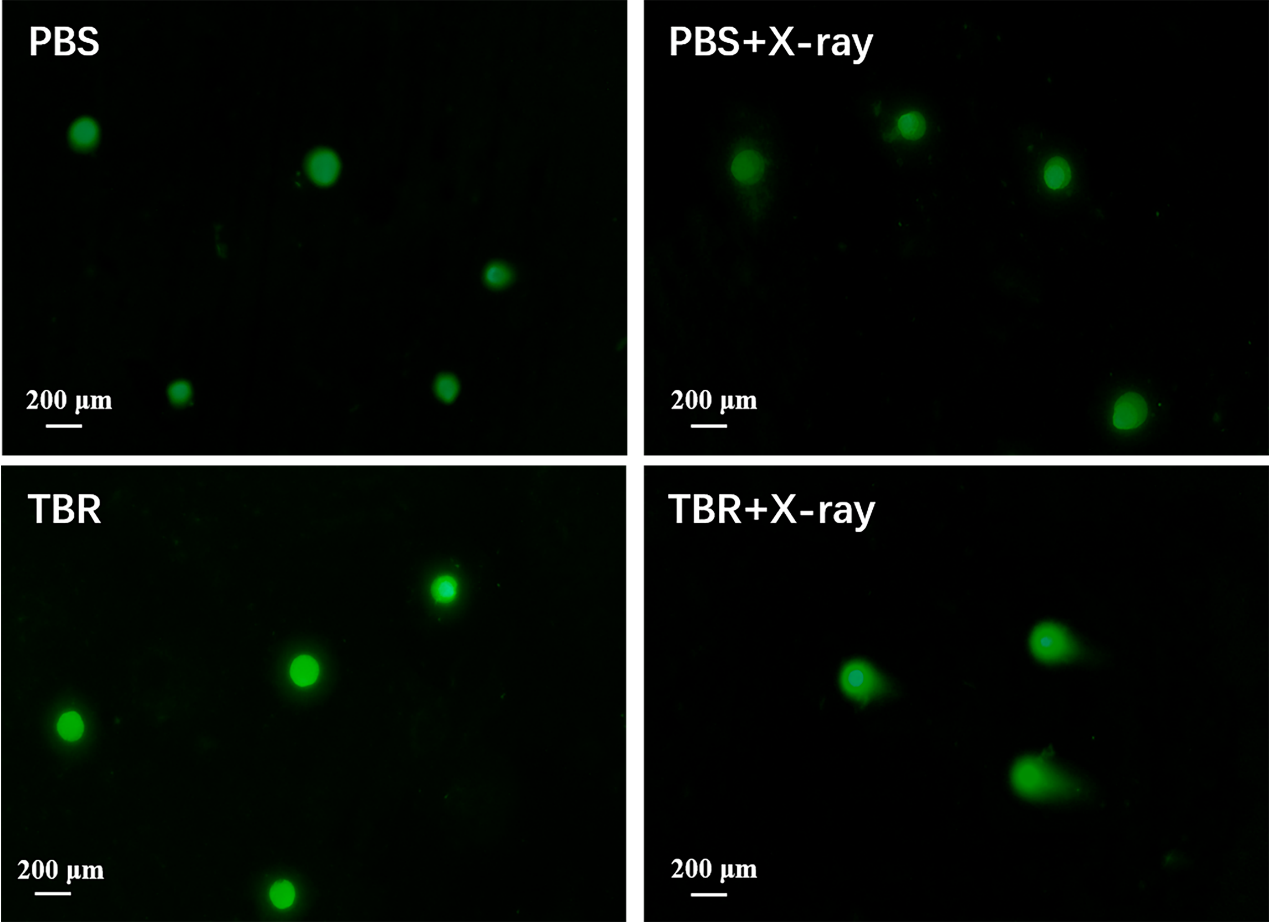


**Figure S10.** Lower magnification images with multiple cells of comet assay.

**
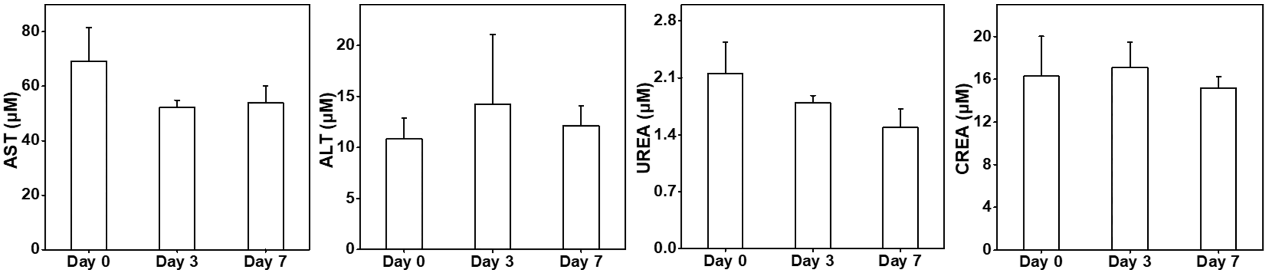
**

**Figure S11.** Mice were intravenously treated daily for 3 days with TBR (20 mg/kg). Blood samples were collected for serum chemistry analysis before treatment (day 0), and at day 3 and day 7 post- intravenous treatment. AST, aspartate transaminase; ALT, alanine transaminase; UREA, blood urea nitrogen; CREA, creatinine.

**
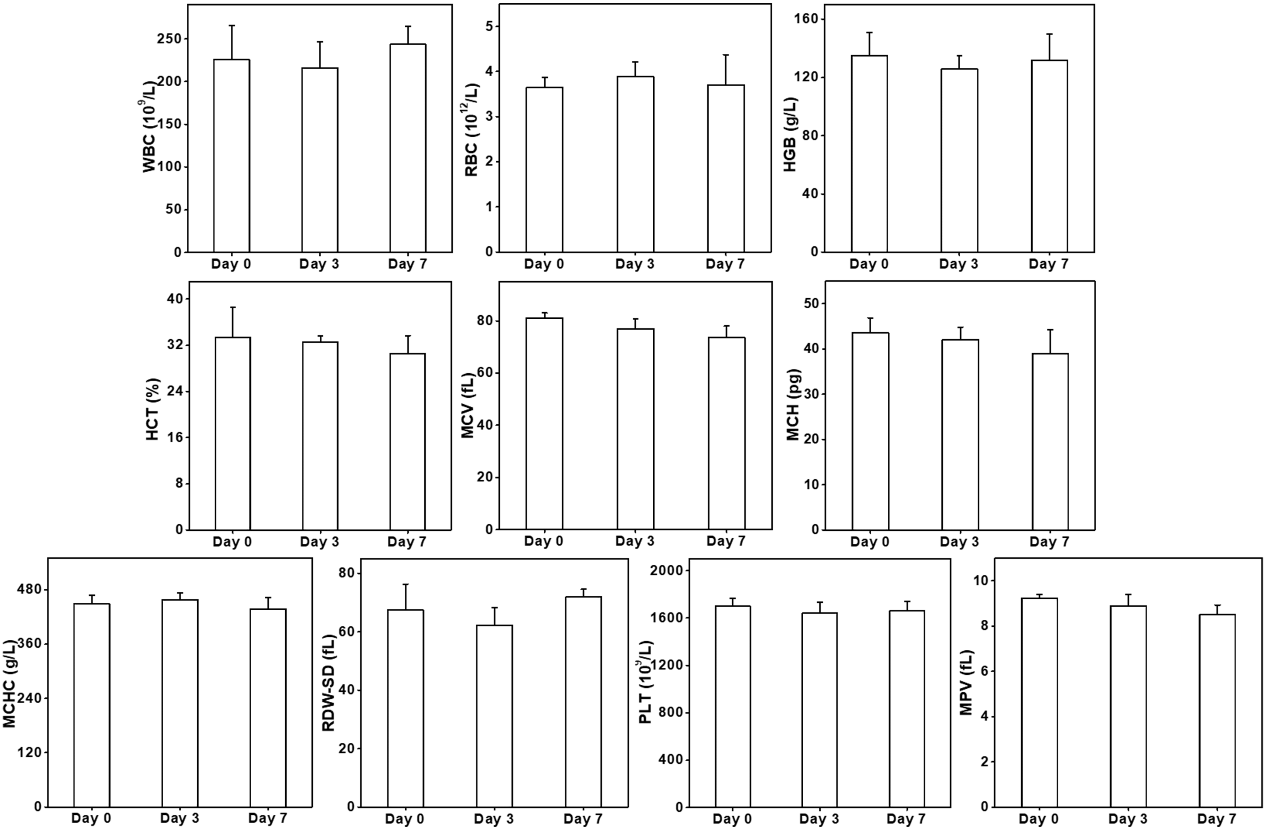
**

**Figure S12.** Mice were intravenously treated daily for 3 days with PBS or TBR (50 mg/kg). Blood samples were collected for complete blood analysis before treatment (day 0), and at day 3 and day 7 post- intravenous treatment. WBC, white blood cell; RBC, red blood cell; HGB, hemoglobin; HCT, hematocrit; MCV, mean corpuscular volume; MCH, mean corpuscular hemoglobin; MCHC, mean corpuscular hemoglobin concentration; RDW-SD, RBC distribution width; PLT, platelets; MPV, mean platelet volume.

**
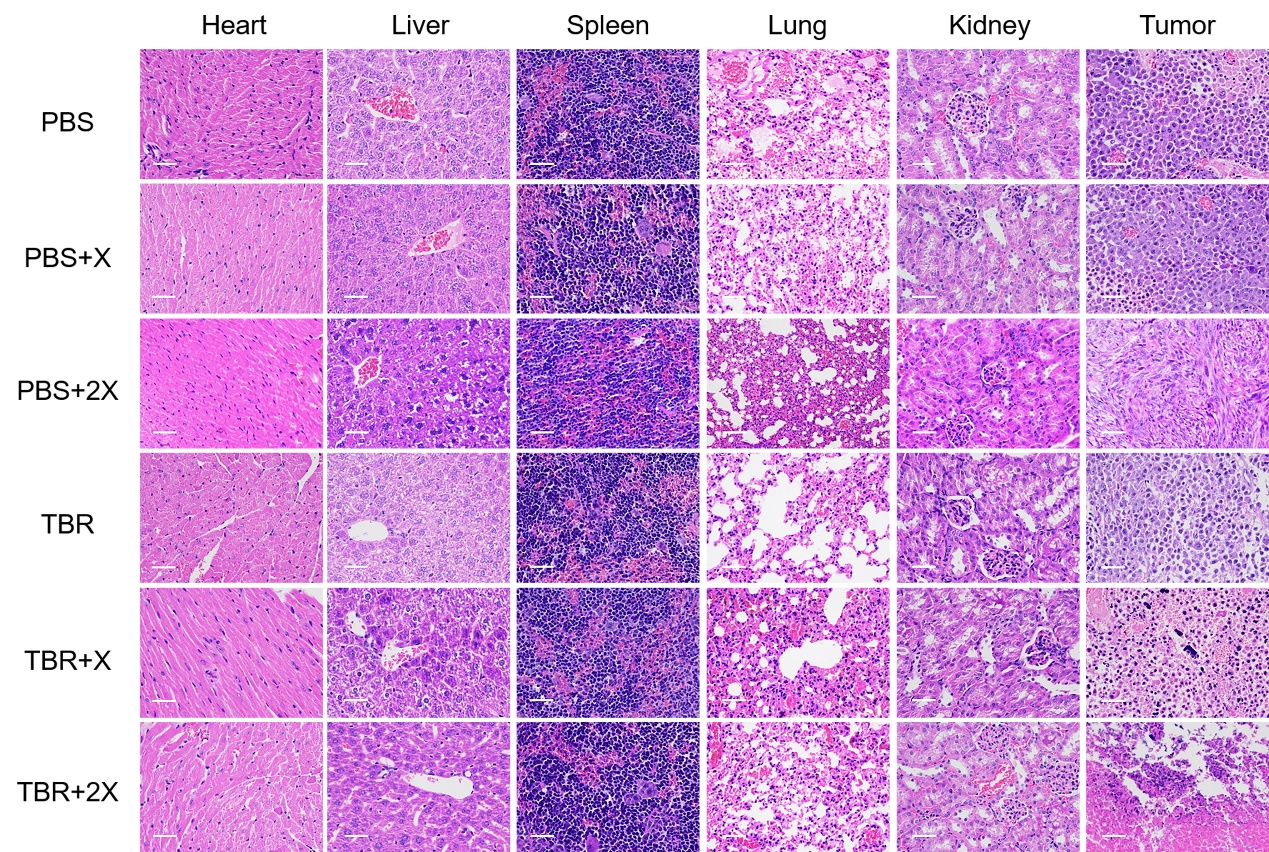
**

**Figure S13.** H&E staining of main organs of mice after different treatments. (Scale bar: 100 µm)


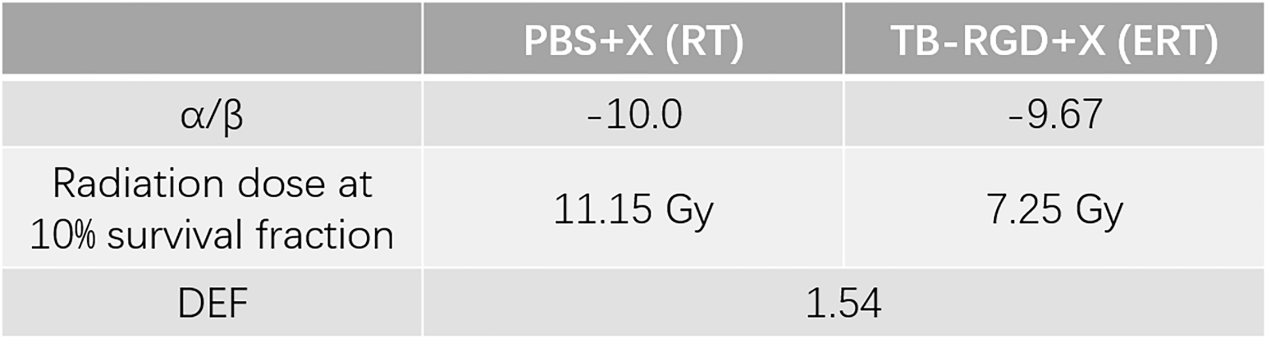


**Table S1.** Radiation enhancement related factor values of TBR by clonogenic assay

**
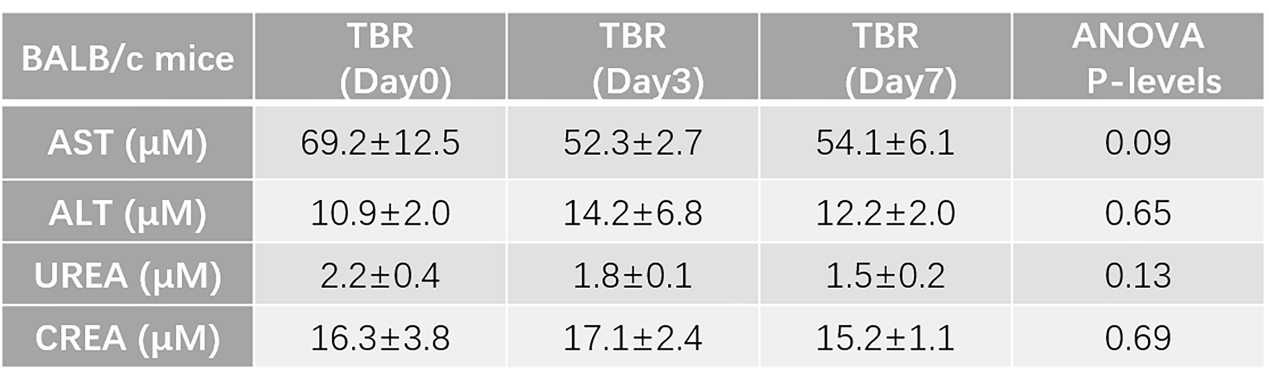
**

**Table S2.** Serum chemistry of mice after intravenous injection with TBR. Data are mean ± s.d.

**
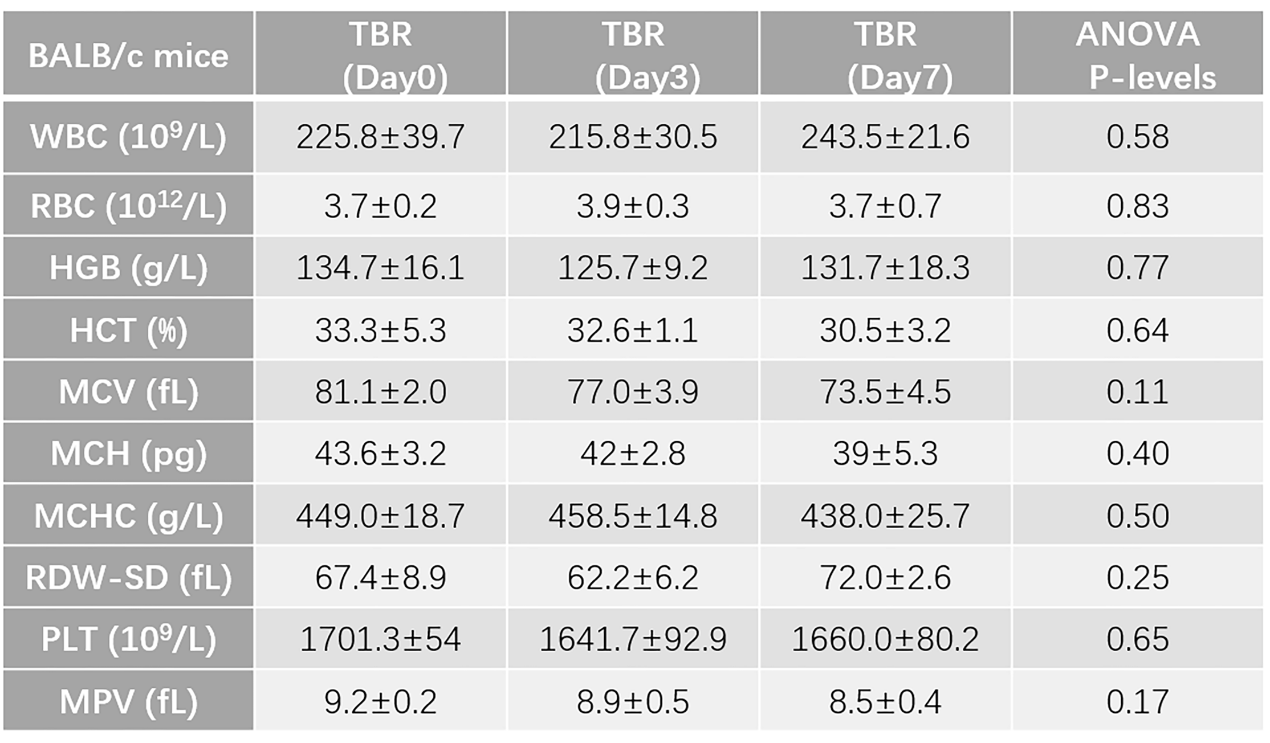
**

**Table S3.** Complete blood count of mice after intravenous injection with TBR. Data are mean ± s.d
